# Nature-Inspired Gallinamides Are Potent Antischistosomal Agents: Inhibition of the Cathepsin B1 Protease Target and Binding Mode Analysis

**DOI:** 10.1021/acsinfecdis.3c00589

**Published:** 2024-05-17

**Authors:** Petra Spiwoková, Martin Horn, Jindřich Fanfrlík, Adéla Jílková, Pavla Fajtová, Adrian Leontovyč, Radka Houštecká, Lucia Bieliková, Jiří Brynda, Marta Chanová, Helena Mertlíková-Kaiserová, Eduardo J. E. Caro-Diaz, Jehad Almaliti, Nelly El-Sakkary, William H. Gerwick, Conor R. Caffrey, Michael Mareš

**Affiliations:** † Institute of Organic Chemistry and Biochemistry of the Czech Academy of Sciences, Flemingovo n. 2, Prague 6 16610, Czech Republic; ‡ Department of Biochemistry and Microbiology, University of Chemistry and Technology, Technická 5, Prague 6 16628, Czech Republic; § Center for Discovery and Innovation in Parasitic Diseases, Skaggs School of Pharmacy and Pharmaceutical Sciences, University of California, La Jolla, San Diego, California 92093, United States; ∥ First Faculty of Medicine, Charles University, Kateřinská 32, Praha 2 12108, Czech Republic; ⊥ Institute of Immunology and Microbiology, First Faculty of Medicine, Charles University and General University Hospital in Prague, Studničkova 2028/7, Prague 2 12800, Czech Republic; # Scripps Institution of Oceanography, University of California, La Jolla, San Diego, California 92093, United States

**Keywords:** cathepsin B, cysteine protease, drug target, parasite, *Schistosoma mansoni*, acrylamide inhibitor

## Abstract

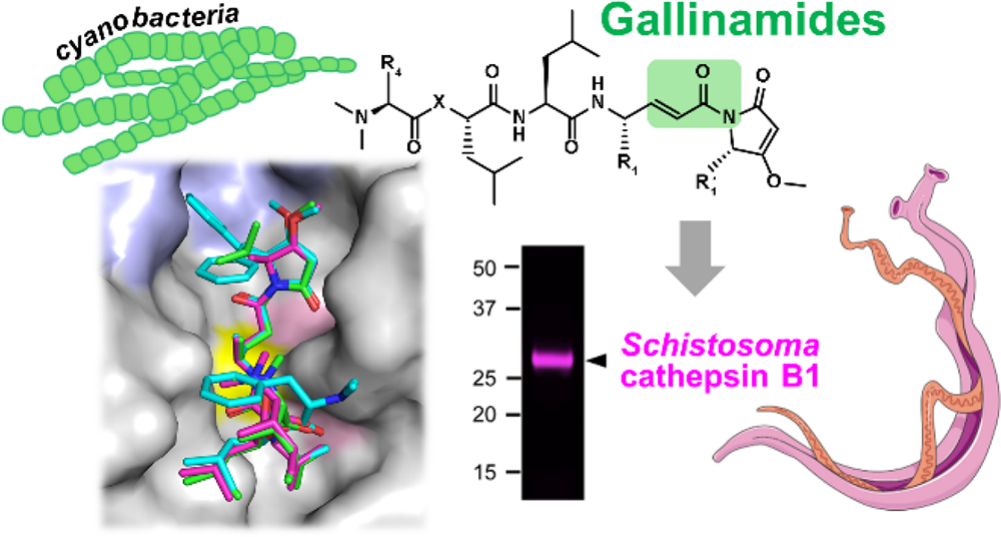

Schistosomiasis, caused by a parasitic blood fluke of the genus *Schistosoma,* is a global health problem for which new chemotherapeutic options are needed. We explored the scaffold of gallinamide A, a natural peptidic metabolite of marine cyanobacteria that has previously been shown to inhibit cathepsin L-type proteases. We screened a library of 19 synthetic gallinamide A analogs and identified nanomolar inhibitors of the cathepsin B-type protease SmCB1, which is a drug target for the treatment of schistosomiasis mansoni. Against cultured *S. mansoni* schistosomula and adult worms, many of the gallinamides generated a range of deleterious phenotypic responses. Imaging with a fluorescent-activity-based probe derived from gallinamide A demonstrated that SmCB1 is the primary target for gallinamides in the parasite. Furthermore, we solved the high-resolution crystal structures of SmCB1 in complex with gallinamide A and its two analogs and describe the acrylamide covalent warhead and binding mode in the active site. Quantum chemical calculations evaluated the contribution of individual positions in the peptidomimetic scaffold to the inhibition of the target and demonstrated the importance of the P1′ and P2 positions. Our study introduces gallinamides as a powerful chemotype that can be exploited for the development of novel antischistosomal chemotherapeutics.

Schistosomiasis is a chronic parasitic disease caused by a trematode blood fluke of the genus *Schistosoma*. According to the WHO, more than 250 million people in the tropics and subtropics required treatment in 2021.^1^ Morbidity associated with the disease arises from immunopathological reactions to parasite eggs that accumulate in different tissues depending on the *Schistosoma* species.^2,3^ This can result in chronic pain and malaise that may interfere with academic performance in school and the ability to perform manual labor, which, among other more life-threatening morbidities, limits the economic productivity of the subsistence communities affected.^4,5^ Treatment and control of disease rely on the use of a single drug, praziquantel, which acts on a calcium-permeable ion channel.^6,7^ The drug has a number of pharmacological and pharmaceutical drawbacks, including poor efficacy against developing schistosomes, and with the concern of resistance, there is a need to identify novel antischistosomal drugs.^8−10^

Adult schistosomes live in the venous blood system where host blood proteins are important source of nutrients for their growth, development, and reproduction. In the schistosome gut, a network of cysteine and aspartic proteases digests host proteins into absorbable peptides and amino acids.^11,12^ The component digestive proteases in *Schistosoma mansoni* (*S. mansoni*) include the CA clan cysteine proteases, cathepsin B1, cathepsin C (dipeptidyl aminopeptidase I), and cathepsins L1–L3, the CD clan protease known as legumain (asparaginyl endopeptidase), and the clan AA aspartic protease, cathepsin D.^11,13,14^*S. mansoni* cathepsin B1 (SmCB1) is a central digestive protease due to its high abundance and dual mode of action as both an endo- and an exopeptidase (specifically, a carboxydipeptidase).^15,16^ This dual activity is enabled by the flexible occluding loop that restricts access to the active site.^15^ The occluding loop is characteristic of cathepsin B-type proteases and distinguishes them from cathepsin L-type proteases, which are strict endopeptidases.^17^ SmCB1 has been validated as a chemotherapeutic target in a murine model of *S. mansoni* infection using a peptidyl vinyl sulfone protease inhibitor.^18^ Subsequent studies with cultured schistosomes identified potent peptidomimetic inhibitors of SmCB1 that possess vinyl sulfone and azanitrile warheads for covalent interaction with the catalytic cysteine, thus inactivating the enzyme.^15,19−21^ The data arising stimulated the search for additional inhibitor chemotypes with improved potency, bioactivity, and metabolic stability. In this context, we explored gallinamides, peptidomimetics with an electrophilic acrylamide warhead, which are derived from the natural product, gallinamide A. Also known as symplostatin 4, gallinamide A is a secondary metabolite found in the *Schizothrix* spp. and *Symploca* spp. marine cyanobacteria that was independently discovered by two groups.^22,23^ The backbone of gallinamide A is a depsipeptide, wherein one N-terminal peptide bond is replaced by an ester bond (Figure 1). The inhibitor contains one leucine residue and four modified amino acids. An unusual 4-(*S*)-amino-2-(*E*)-pentenoyl moiety is a major part of the reactive acrylamide warhead belonging to the α,β-unsaturated Michael acceptors, which interact with cysteine proteases in a covalent, irreversible manner.^24^ It is followed at the C-terminus by a methylmethoxypyrrolinone (MMP) moiety, contributing to the inhibitor reactivity.^25^ Gallinamides inhibit several cathepsin L-type cysteine proteases, including the falcipains from *Plasmodium falciparum*, and cruzain from *Trypanosoma cruzi*, and were antiparasitic *in vitro*.^22,24,26−28^ Gallinamides also inhibit human cathepsin L and suppress cathepsin L-mediated cell infection by SARS-CoV-2.^28,29^

**Figure 1:**
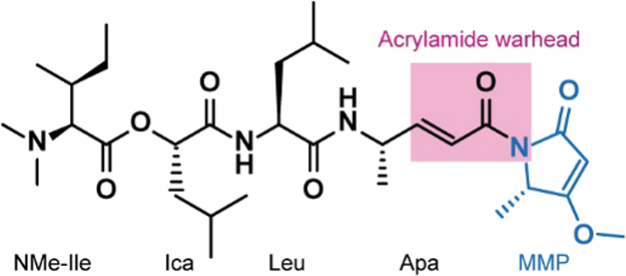
Chemical structure of gallinamide A. The acrylamide warhead is boxed in pink, and the C-terminal methylmethoxypyrrolinone (MMP) moiety is shown in blue. The N-terminal part of the molecule contains *N*,*N*-dimethyl-l-isoleucine (NMe-Ile), followed by isocaproic acid (Ica), leucine (Leu), and 4-(*S*)-amino-2-(*E*)-pentenoic acid (Apa).

Here, we report gallinamides as a nature-inspired chemotype that yields novel, highly potent antischistosomal compounds. We show that the bioactivity of gallinamides is associated with the inhibition of SmCB1, a cathepsin B-type cysteine protease. We define a structure–activity relationship for gallinamides with SmCB1, solve cocrystal complexes between SmCB1 and representative gallinamides, and employ quantum chemical calculations to describe the inhibitory interactions with the target. Together, the data provide a valuable basis for the further rational design of novel chemotherapeutic agents to treat schistosomiasis.

## Results

### Gallinamide A Inhibits Cathepsin B Activity in the Schistosome Parasite

We investigated whether gallinamide A inhibits the activity of the major proteases associated with proteolytic digestion in the adult schistosome gut. The digestive protease network includes the cysteine proteases cathepsins B, C, L from clan CA and legumain from clan CD, and an aspartic protease cathepsin D from clan AA.^11,12^ Their respective activities can be measured in protein extracts of *S. mansoni* using a kinetic assay with specific fluorogenic substrates. In addition, these activities can be attributed to particular proteases based on sensitivity to selective inhibitors^30−32^ (Table 1). Accordingly, we exposed worm extracts to gallinamide A and analyzed the individual protease activities. Gallinamide A inhibited both the carboxydipeptidase (98%) and endopeptidase activities (94%) of cathepsin B as measured with the respective substrates, Abz–Phe–Arg–Val–Nph^15^ and Cbz–Arg–Arg–AMC^33,34^ (Table 1). The activity measured with the Cbz–Phe–Arg–AMC substrate,^11,13,16,33,35^ which can be cleaved by both cathepsins B and L, was inhibited by gallinamide A to a lesser extent (72%). The inhibitor had little to no effect (<10% inhibition) on the activities of cathepsin C, legumain, or cathepsin D.

**Table 1:** Inhibition of Digestive Protease Activities of *S. mansoni* Extract by Gallinamide A

**target protease**	**proteaseclass/clan/family**^a^	**cleavage mode**	**substrate**^b^	**inhibition (%)**^c^
cathepsin B	cysteine/CA/C1	exopeptidase (carboxydipeptidase)	Abz–Phe–Arg–Val–Nph	98.0 ± 2.2
cathepsin B	cysteine/CA/C1	endopeptidase	Cbz–Arg–Arg–AMC	93.7 ± 0.3
cathepsins B and L	cysteine/CA/C1	endopeptidase	Cbz–Phe–Arg–AMC	71.9 ± 2.0
cathepsin C	cysteine/CA/C1	exopeptidase (aminodipeptidase)	Gly–Arg–AMC	2.8 ± 1.9
legumain	cysteine/CD/C13	endopeptidase	Cbz–Ala–Ala–Asn–AMC^d^	8.2 ± 0.1
cathepsin D	aspartic/AA/A1	endopeptidase	Abz–Lys–Pro–Ala–Glu–Phe–Nph–Ala–Leu^d^	0.01 ± 1.54

a Classification according to the MEROPS database.^36^

b Individual protease activities in the extract of adult *S. mansoni* were measured with specific peptide substrates using a continuous fluorometric assay. In addition, these activities were found to be sensitive to diagnostic inhibitors of target proteases,^30,31^ including CA-074 for cathepsin B, E-64 for cathepsins B and L, Ala–Hph–VS–Ph for cathepsin C, Aza-N-11a for legumain, and pepstatin for cathepsin D (see Methods for details), which provided >95% inhibition.

c Measured with 1 μM gallinamide A; values are expressed as percentage of inhibition relative to the uninhibited control. Means ± SD of triplicates are given.

d Measured in the presence of E-64 to prevent interference from the activity of potentially cross-reacting cysteine cathepsins,^30,31^ as described in Methods.

In conclusion, the inhibition by gallinamide A of the various protease activities in the *S. mansoni* extract indicated that cathepsin B was inhibited. The cathepsin B activity measurable is largely due to SmCB1, the most abundant digestive protease of *S. mansoni*.^11,16,32^ Therefore, we next characterized the functional and structural interactions of gallinamides with SmCB1.

### SAR Analysis of Gallinamide A Analogs with SmCB1

Gallinamide A and a series of its 18 derivatives were evaluated *in vitro* as inhibitors of SmCB1 to explore the binding specificities of the subsites in the enzyme’s active site. The gallinamide A inhibitor scaffold can be defined by positions P4 through P1′ (Schechter and Berger nomenclature^37^), which were assigned according to the binding mode analysis (see the crystallographic section below). The substituents R_4_, R_1_, and R_1_′ introduced on the scaffold (Table 2) are numbered according to the respective positions P4, P1, and P1′. The structural relationships among the investigated compounds are listed in Table 2. The compounds’ second-order rate constants (*k*_2nd_) were determined using a kinetic inhibition assay with recombinant SmCB1 and the substrate, Cbz–Phe–Arg–AMC. The identified one-step kinetic mechanism does not allow for the determination of the individual parameters of *k*_inact_ and *K*_*i*_, but only the cumulative *k*_2nd_ = *k*_inact_/*K*_*i*_.^38^

**Table 2:**
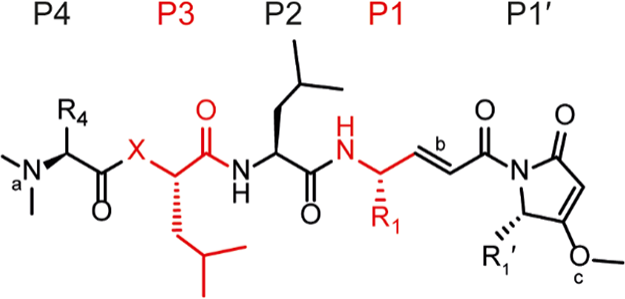
Inhibition of SmCB1 and the Antischistosomal Activity of Gallinamide A and Its Derivatives

						schistosomula severity score^f^		
	substituent position^d^				SmCB1 inhibition^e^	time (h)		
compound	R_4_	X	R_1_	R_1_′	*k*_2nd_ (M^–1^·s^–1^)	24	48	72
gallinamide A	Ile	O	Ala	Ala	6644 ± 532	1	2	4
**1**	Ile	O	Ala	Leu	3394 ± 133	2	4	4
**2**	Val	O	Ala	Ala	3334 ± 559	0	1	4
**3**^a^	Ile	N	Ala	Ala	2902 ± 172	0	0	2
**4**	Ile	N	Ala	Ala	2894 ± 113	0	0	4
**5**^c^	Ile	O	Ala	Ala	2792 ± 360	0	4	4
**6**	Phe	O	Ala	Phe	1930 ± 304	2	4	4
**7**	Ile	O	Phe	R-Phe	942 ± 50	0	3	4
**8**	Ile	O	Phe	Ala	887 ± 46	1	4	4
**9**	Ile	O	Ala	Phe	793 ± 75	1	2	4
**10**	Ile	O	Phe	Leu	537 ± 48	0	2	4
**11**	Val	O	Phe	Leu	295 ± 17	1	3	4
**12**	Phe	O	Phe	Ala	212 ± 30	1	2	4
**13**	Phe	O	Phe	R-Phe	109 ± 2	0	2	4
**14**	Ile	O	Phe	Phe	54.5 ± 1.3	0	3	4
**15**	Ile	O	R-Phe	R-Phe	41.6 ± 1.6	0	3	4
**16**^b^	Ile	O	Ala	Ala	36.9 ± 0.3	0	0	0
**17**	Phe	O	Phe	Phe	18.2 ± 0.2	0	0	4
**18**	Ile	O	R-Phe	Phe	16.0 ± 0.2	0	0	0

a *N*,*N*-Dimethylamine group substituted by the *N*-acetamide group (CH_3_–CO–NH−) in **3**.

b Compound **16** without the double bond in the acrylamide warhead.

c Methoxy group on the MMP ring substituted by the *O*-hexynyl group (−O–(CH_2_)_4_–C≡CH) in **5**.

d The gallinamide structures are defined by the compound core (see scheme) and the substituents R_4_ to R_1_′, numbered according to positions P4–P1′ (see the crystallographic section).

e The second-order rate constants *k*_2nd_ were measured in a kinetic activity assay with the fluorogenic peptide substrate Cbz–Phe–Arg–AMC. Mean ± SE values are given. Inhibitors are ranked according to their *k*_2nd_ values.

f Phenotypic changes in newly transformed schistosomula (NTS) of *S. mansoni* induced by 1 μM compound were recorded at three time points and converted to a severity score on a scale from 0 (no effect) to 4 (the most severe) as described previously;^19,20,39^ data are shown as a heat map. The full data set is presented in Table S1. Compounds were tested in duplicate in two independent assays, and representative data are shown.

Gallinamide A was identified as the most potent inhibitor of SmCB1 among the compounds tested with a *k*_2nd_ value of 6644 M^–1^ s^–1^. The critical role of the reactive warhead for inhibition was demonstrated by **16** in which the absence of a double bond in the acrylamide group led to a dramatic decrease in *k*_2nd_ by 2 orders of magnitude (Table 2).

A series of substitutions at R_4_, R_1_, and R_1_′ were made in the gallinamide A molecule (Table 2). The N-terminal *N*,*N*-dimethyl-Ile residue in gallinamide A is linked to the rest of the inhibitor by an ester bond, and the latter’s replacement by an amide bond resulted in a 2.3-fold decrease in inhibitory potency, as shown for **3** and **4**. Both of these analogs differ in the N-terminal groups of dimethylamine (**4**) and acetamide (**3**), yet they exhibit similar inhibitory activity. Gallinamide A contains a C-terminal MMP moiety. Extension of the methoxy group on the MMP ring to the *O*-hexynyl moiety (−O–(CH_2_)_4_–C≡CH) in **5** reduced the inhibitory potency by 2.4-fold. Substitutions in the terminal R_4_ and R_1_′ (Ile and Ala in gallinamide A) with other aliphatic residues (Val and Leu) in **1** and **2** resulted in only a 2-fold decrease in inhibitor potency; this effect is also seen for another set of analogs **8**, **10**, and **11** (vs **8**). Incorporation of larger aromatic residues represented by Phe decreased the inhibition potency of compounds **6** and **9** by 3.4 and 8.4 times, respectively. This decrease is even stronger for **8**, **12**, **14**, and **17**, with a 4.2–49-fold decrease (vs **8**), which carry also an additional Phe as R_1_. Substitution of R_1_ Ala (gallinamide A) with Phe resulted in a 7.5-fold decrease in the inhibitor potency of **8**. Changing the stereochemistry of Phe in R_1_ from the S to R configuration significantly decreased inhibition: 23-fold for **15** (vs **7**) and 3.4-fold for **18** (vs **14**). In contrast, changing the configuration of Phe in R_1_′ from S to R improved the inhibition: 17-fold for **7** (vs **14**), 6-fold for **13** (vs **17**), and 2.6-fold for **15** (vs **18**).

In summary, we characterized the inhibition of SmCB1 with gallinamide A and six analogs, which generated *k*_2nd_ values >10^3^ M^–1^·s^–1^. The analysis provided initial information on the affinities of the subsites S4, S1, and S1′ of SmCB1. In particular, a cumulative effect of aromatic substitutions and their configuration in the P1 and P1′ positions were found to significantly influence the potency of gallinamides targeting SmCB1.

### Gallinamides Are Potent Antischistosomal Compounds

The same 19 gallinamides were screened for bioactivity against *S. mansoni* newly transformed schistosomula (NTS), the postinvasive parasite stage that feeds on host blood.^40^ In this primary assay, the NTS were exposed to 1 and 10 μM compounds for 3 days, and the resulting phenotypic responses were graded from 0 through 4, i.e., the least to the most severe (Table 2 and Table S1). Fourteen of the 19 compounds tested induced phenotypic changes at 1 μM after 48 h, and of these, seven had a strong effect (grade 3 or 4; Table 2). The effects were more pronounced after 72 h, and when the higher, 10 μM, compound concentration was used (Table S1). The weakest bioactivity was displayed by (i) compound **16**, which lacks the reactive warhead, (ii) **17** and **18**, which are weak inhibitors of SmCB1, and (iii) **3** and **4**, which have an amide, rather than an ester, bond at P3. Also, compared to gallinamide A and its analogs, **3** has an additional N-terminal *N*-acetamide modification that further reduces bioactivity (Table 2). In this context, it is noteworthy that **3** and **4** have the lowest logP and highest TPSA values (Table S2), suggesting their low permeability, which is consistent with previous reports comparing the permeability of peptides containing ester and amide linkages.^41^

In the next step, the bioactive compounds gallinamide A, **1**, **6**, and **9** (selected for structural analyses (see below)) were phenotypically screened against adult schistosomes. Experiments were performed at 1, 2, 5, and 10 μM with *ex vivo* adult mixed sex *S. mansoni*, and the phenotypes observed over 48 h were assigned severity scores ranging from 0 to 4 (Table 3 and Table S3). Compounds **6** and **9**, followed by **1**, were the most potent as a function of time and concentration, generating severity scores of 4 after 24 h at 10 μM (Table 3), which was primarily due to damage of the surface tegument (Table S3).

**Table 3:** Antischistosomal Activity of Gallinamides against *Ex VivoS. mansoni* Adults

	severity scores^a^															
time (h)	2				6				24				48			
compound (μM)	1	2	5	10	1	2	5	10	1	2	5	10	1	2	5	10
gallinamide A	0	0	0	0	0	0	0	0	0	0	0	1	0	2	2	3
**1**	0	0	0	0	0	0	0	1	0	0	0	4	0	2	2	4
**6**	0	0	1	2	0	0	1	2	0	0	2	4	0	1	4	4
**9**	0	0	1	1	0	0	1	2	0	0	2	4	0	2	4	4

a Phenotypic changes in adult *S. mansoni* induced by compounds at given concentrations were observed and converted to severity scores on a scale of 0 (no effect) to 4 (severe effects) as described previously.^39,42,43^ Data are shown as a heat map. The full phenotypic details are presented in Table S3. Compounds were tested in duplicate in two independent assays, and representative data are shown.

In conclusion, many gallinamides possess potent bioactivity against two developmental stages of *S. mansoni*. The induced phenotypic changes were parasite-specific, as suggested by the good cytotoxicity profile recorded with several human cell lines for the prototype compound, gallinamide A, and three analogs (Table S4).

### SmCB1 Is a Target for Gallinamides

**Figure 2:**
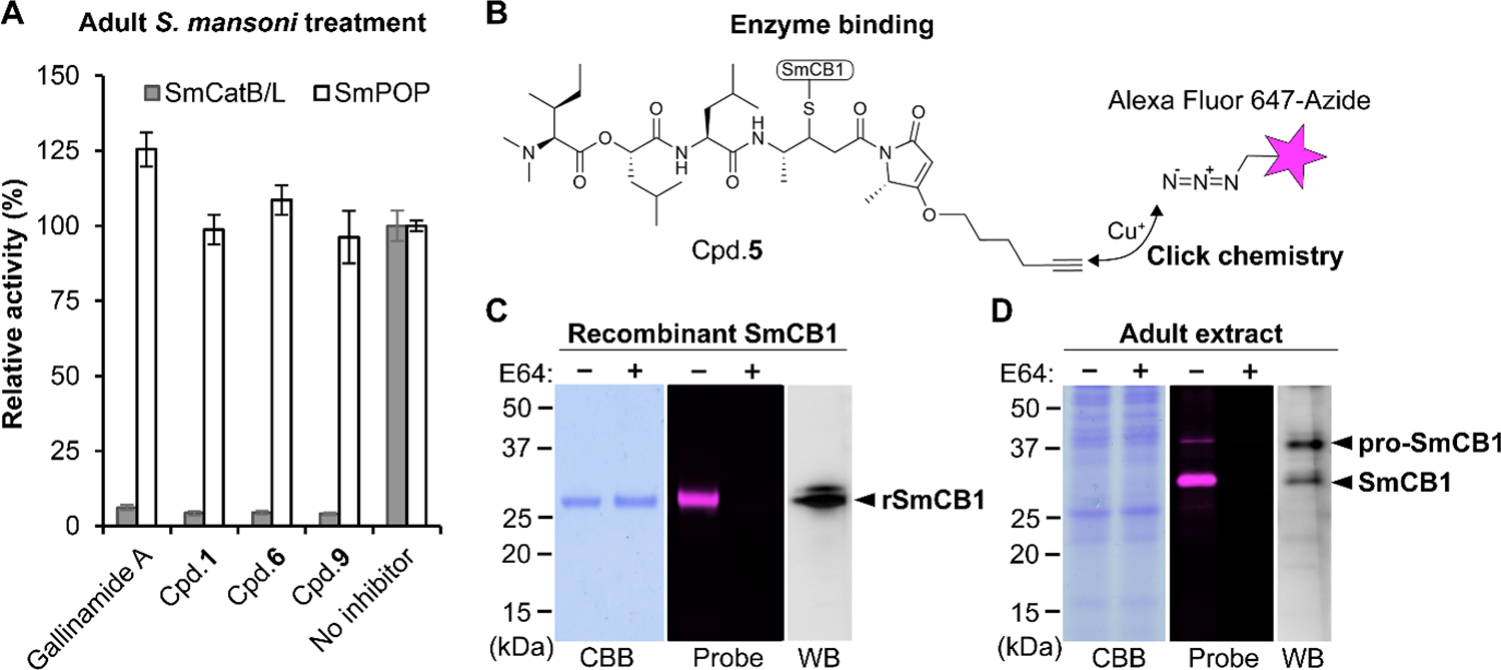
SmCB1 is a target for gallinamides in adult *S. mansoni*. (A) Mixed sex, adult *S. mansoni* were incubated for 48 h with 5 μM gallinamide A or **1**, **6**, and **9**. After washing and preparation of worm extract, proteolytic activities (0.2 μg of protein) were measured in a kinetic assay using the cathepsin B and L substrate, Cbz–Phe–Arg–AMC (SmCatB/L, gray bars) at pH 5.5 or the prolyl oligopeptidase substrate, Cbz–Gly–Pro–AMC (SmPOP, white bars) at pH 8.0. Means ± SE of three replicates are normalized to the control not exposed to the inhibitor (set as 100%). (B–D) Imaging of SmCB1 using the gallinamide compound **5** as an activity-based probe. **5** contains an alkyne tag that can be modified by a copper-catalyzed click reaction with a fluorescent reagent (Alexa Fluor 647-azide) (B). Recombinant SmCB1 (rSmCB1, 0.5 μg) (C) or extracts of adult *S. mansoni* worms (7 μg of protein) (D) were incubated with **5** (10 μM) for 1 h and then clicked. Reactions were resolved by SDS-PAGE, and gels were visualized using a fluorescence scanner (Probe), stained for protein using Coomassie Brilliant Blue (CBB), or analyzed by Western blot with an anti-SmCB1 antibody (WB). In a control experiment, rSmCB1 or worm extract was treated with the competitive protease inhibitor E-64 (10 μM), prior to incubation with **5**. The positions of rSmCB1, native SmCB1, and its pro-form (pro-SmCB1) are indicated. The mass difference between rSmCB1 and native SmCB1 is caused by glycosylation (see Methods). The active site of the pro-SmCB1 is shielded by a propeptide^45^ that prevents effective binding of **5**.

Adult mixed sex *S. mansoni* were incubated in the presence of 5 μM gallinamide A, **1**, **6**, or **9** for 24 h, and protein extracts therefrom were assayed with the cathepsins B and L substrate, Cbz–Phe–Arg–AMC. Protease activity was reduced by ∼95% relative to the activity of untreated controls (Figure 2A). For comparison, we also quantified the activity of the nondigestive proteolytic enzyme, *S. mansoni* prolyl oligopeptidase (SmPOP). This serine protease, which is localized in the tegument of adult worms, was assayed with the selective substrate Cbz–Gly–Pro–AMC.^44^ SmPOP was unaffected by the gallinamide treatment.

To directly demonstrate the molecular target(s) of the gallinamides in the adult *S. mansoni*, we designed an activity-based probe (ABP) based on the alkyne-tagged gallinamide analog **5** (Table 2) and visualized binding by the probe to the enzyme target via SDS-PAGE (Figure 2B–D). The alkyne tag on **5** allows for the conjugation of a fluorescent reagent (such as Alexa Fluor 647-azide) by click chemistry using a copper-catalyzed azide–alkyne cycloaddition (Figure 2B). Recombinant SmCB1 or worm extract was incubated with **5**, then modified by the click reaction, and subjected to SDS-PAGE and fluorescence imaging. A fluorescent band at ∼30 kDa corresponding to the SmCB1–**5** adduct was observed for both the recombinant SmCB1 (Figure 2C) and worm extracts (Figure 2D). In the latter, the pro-form of SmCB1 was also visualized at ∼37 kDa as authenticated by the use of a specific anti-SmCB1 antibody and immunoblotting. For both the recombinant enzyme and worm extracts, the binding of **5** to the target was blocked by prior incubation with the competitive cysteine protease inhibitor, E-64. Thus, ABP-**5** is a useful tool for visualizing SmCB1, including in complex worm extracts.

In conclusion, biochemical analysis of adult *S. mansoni* worms demonstrated that cathepsin B activity is suppressed in worms exposed to gallinamides and that SmCB1 is recognized by a gallinamide-derived reactive probe. Overall, SmCB1 is a major target for gallinamide antischistosomal agents.

### Crystallographic Analysis of the Binding Mode of Gallinamide Inhibitors with SmCB1

#### Crystal Structures of Three SmCB1–Gallinamide Complexes

**Figure 3:**
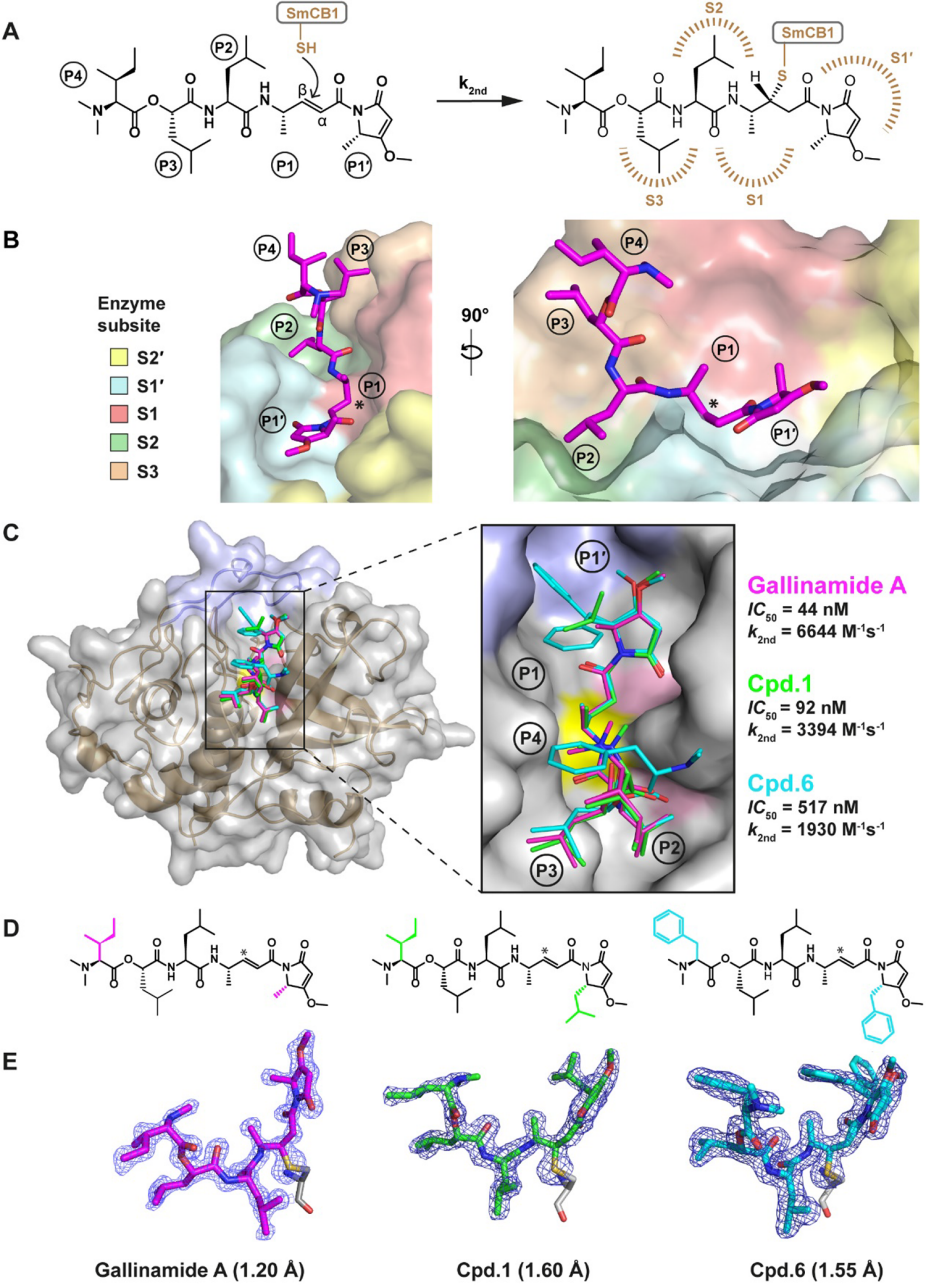
Binding mode of gallinamide inhibitors in the SmCB1 active site. (A) Reaction scheme of gallinamide A forming an irreversible covalent bond with the thiol of the catalytic cysteine residue of SmCB1 (brown). In the acrylamide warhead, C atoms of the reactive vinyl group are labeled (α, β). Individual positions (P) of the inhibitor and binding subsites (S) of the enzyme are indicated. (B) Conformation of gallinamide A (magenta sticks) in the SmCB1 active site (surface). The binding subsites S3 to S1′ (colored) interact with the corresponding inhibitor positions P3–P1′. The P4 position is oriented out of the active site; the S2′ subsite is not directly occupied. (C) Overall crystal structure of three SmCB1 complexes with gallinamide A and analogs **1** and **6**. The enzyme is shown as a gray surface (with a cartoon embedded), containing the occluding loop in blue and the catalytic cysteine and histidine residues in yellow and pink, respectively. Inhibitors in sticks representation are color coded for C atoms (heteroatoms are colored red and blue for O and N, respectively). The zoomed in view of the SmCB1 active site shows a superposition of the inhibitors (note two different P1′ orientations in **6**). Inhibition kinetic parameters of the inhibitors against SmCB1 are presented on the right. (D) Chemical structures of gallinamide A, and compounds **1** and **6** with distinguishing substituents highlighted. The Cβ atom forming a covalent bond with SmCB1 is marked with an asterisk. (E) The 2*F*_0_–*F*_c_ electron density maps of the SmCB1–bound inhibitors (colored as in panel C) are contoured at 0.7 σ. The orientation of each inhibitor is set for clarity of presentation, and the covalently bound catalytic cysteine residue (gray) is depicted (without the main-chain map). Crystal structure resolutions are indicated.

Recombinant SmCB1 was crystallized in complex with gallinamide A, and analogs **1** and **6**, which inhibited cathepsin B and displayed bioactivity against both developmental stages of the parasite. All complexes crystallized in the orthorhombic space group *P*2_1_2_1_2_1_ with one molecule in the asymmetric unit and a solvent content of ∼40%. The structures were determined by molecular replacement and refined using data to resolutions of 1.20 1.60, and 1.55 Å for the complexes of gallinamide A, **1**, and **6**, respectively (Table S5). The final crystallographic models contained full-length SmCB1 spanning residues 70–323 (zymogen numbering).^45^ The root-mean-square deviations (RMSDs) for the superposition of the three SmCB1 backbones (Cα atoms) range from 0.10 to 0.12; values that are consistent with RMSDs observed for different crystal structures of the same protein.^19^ The electron density used to model the inhibitors was of good quality (Figure 3E).

#### Binding Mode of Gallinamide Inhibitors with SmCB1

The inhibitors are located in the active site cleft of SmCB1, which contains the catalytic residues Cys100 and His270 (supported by Asn290 and Gln94) and has restricted access to the primed region (beyond S1′ and S2′ subsites) by the occluding loop (Phe175 to Pro197), a hallmark of cathepsins B.^15,17^ Gallinamides form an irreversible covalent bond to the thiol group of Cys100 through the Cß atom of the vinyl group of the acrylamide warhead (Figure 3A). Inhibitor positions P3–P1′ occupy the corresponding binding subsites S3–S1′ of SmCB1. In contrast, the P4 residue of gallinamides points upward, out of the active site cleft (Figure 3B,C). The chemical structures of the three gallinamides are identical at positions P1–P3, while diversity is provided by substitutions at P4 and on the MMP ring at P1′ (Figure 3D). Both terminal positions of the gallinamide scaffold, P4 and P1′, were identified as the most flexible parts of the inhibitors according to the B-factor analysis (Figure S1).

**Figure 4:**
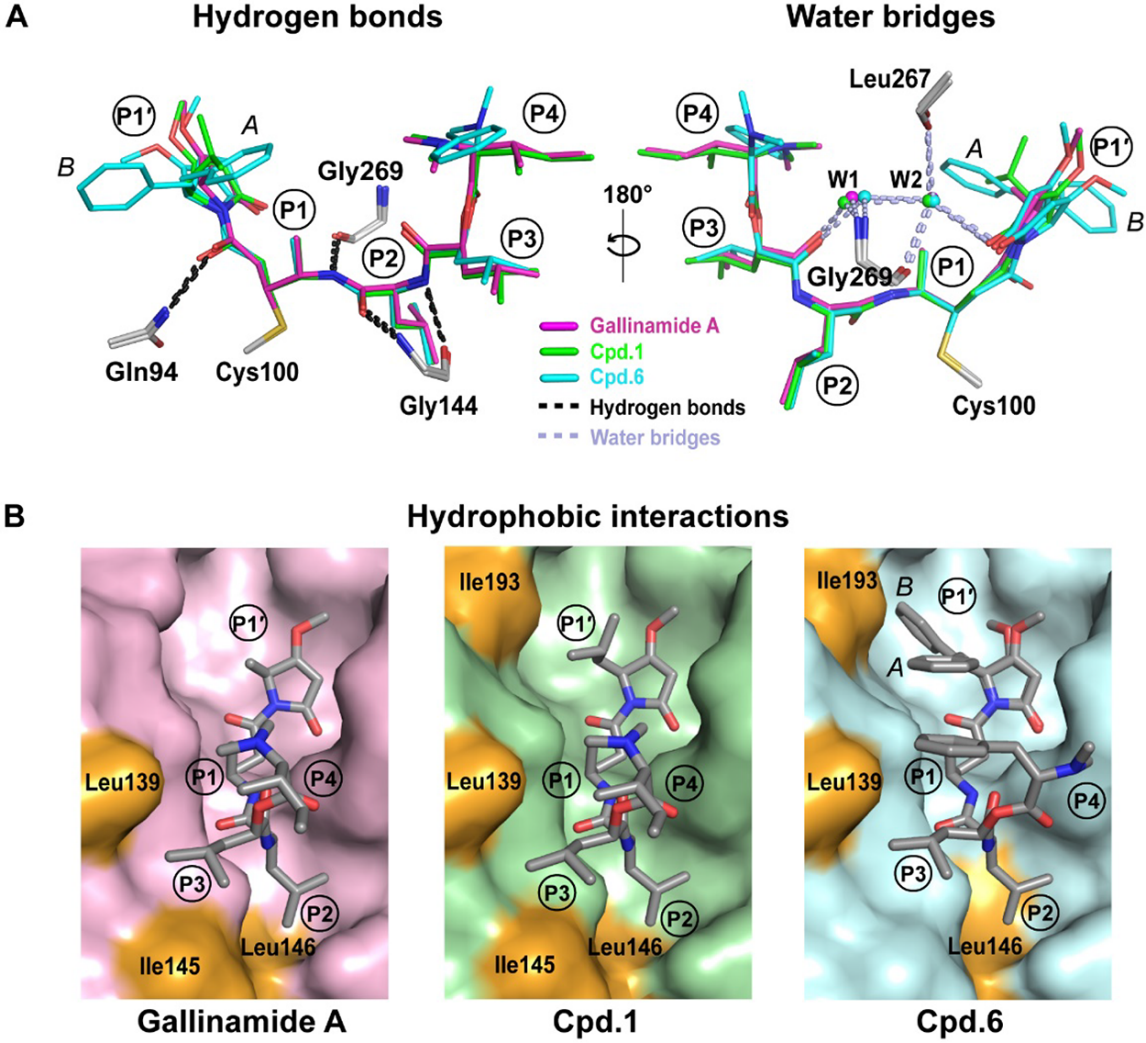
Interaction of gallinamides with active site residues of SmCB1. (A) Left-hand view shows hydrogen bonds (within 3.3 Å distance, dashed black lines) formed between SmCB1 residues (gray) and gallinamide A (magenta), compounds **1** (green) and **6** (cyan). In the right-hand view, indirect polar interactions (within a 3.4 Å distance and with angle criteria taken into account) via two conserved water molecules (W1 and W2 spheres colored as the corresponding inhibitors) are depicted by dashed light blue lines. Heteroatoms have a standard color coding (O, red; N, blue; S, yellow). The P1′–P4 positions of inhibitors are indicated. The inhibitors are covalently bound to catalytic residue Cys100 of SmCB1. (B) Surface representation of the SmCB1 active site. Highlighted in orange are the SmCB1 residues that form hydrophobic interactions (within 4.1 Å distance) with the inhibitors. Inhibitors are shown as gray sticks (heteroatoms are color coded as in panel A). Two different orientations of the P1′ position of **6** are marked. For details on intermolecular contacts and interactions, see Tables S6–S9.

In all complexes, there is a set of common hydrogen bonds between the inhibitor backbone (of P2, P1, and P1′) and the active site residues Gln94, Gly144, and Gly269 (Figure 4A). In addition, two conserved water molecules form a network of indirect polar interactions (intermolecular and intramolecular), bridging the backbones of the P3 and P1′ positions and those of Leu267 and Gly269 residues (Figure 4A). Specific structural determinants of the inhibitors and their interactions in the binding subsites are as follows.

The P1′ position contains the C-terminal MMP group with various R_1_′ substituents, including side chains of alanine, leucine, and phenylalanine in gallinamide A, **1**, and **6**, respectively. Two different conformations were modeled for the phenylalanine side chain in the MMP group of compound **6** (Figures 3C and 4). In one conformation (labeled A in Figure 4), it is flipped out from the S1′ subsite to S1, while in the other (B), it is directed toward the S2′ subsite. The conformation A appears to be intramolecularly stabilized by a CH-π interaction of the P1′-phenylalanine and P1 alanine side chains. Each conformation is associated with a different orientation of the methoxy group on the MMP ring and a different conformation of His181 on the occluding loop in S2′. The spatial relationship of these three structural determinants of **6** upon conformational rearrangement was determined by quantum chemical calculations, as presented in Table S10. It is noteworthy that this is the first report on ligand-induced conformational flexibility of His181 for any cathepsin B, whereby this conserved residue plays an important role in the exopeptidase specificity through recognition of the substrate C-terminus.^15,46,47^ The R_1_′ substituents of the MMP ring form hydrophobic interactions with Ile193 through the leucine and phenylalanine side chains of **1** and **6**, respectively (Figure 4B). A double conformation of Ile193 was observed in the complexes of **1** and gallinamide A, but not in the complex of **6** due to steric hindrance by R_1_′. The core of the MMP ring is not involved in hydrogen/hydrophobic interactions with SmCB1. However, for **6**, we observed a T-shape π–π interaction of the MMP ring in conformation A with Trp292. This interaction was not observed for the other structures analyzed, where the mutual orientation of the MMP and Trp292 is not optimal.

The acrylamide warhead spanning P1′ and P1 contains a carbonyl group that forms the hydrogen bond with the amide group of Gln94 in the “oxyanion hole” of S1 subsite and participates in the water bridge network (Figure 4A). The P1 alanine of the inhibitors forms few contacts within the S1 subsite (Table S6); however, its backbone amide is hydrogen bonded with the Gly269 oxygen (Figure 4A). At the position P2, the inhibitors contain a leucine residue that occupies the S2 subsite of SmCB1, which can accommodate bulky hydrophobic residues.^15^ At the bottom of the S2 pocket, the P2 leucine makes a hydrophobic interaction with Leu146 (Figure 4B) and contacts with Glu316; however, the conformation of Glu316 is not rotated out of the pocket as previously observed for longer and bulky P2 substituents.^19^ The P2 backbone nitrogen and oxygen form two hydrogen bonds with Gly144 (Figure 4A). The P3 position of the gallinamides carries the leucine side chain of the Ica residue (Figure 1). It is accommodated in a wide and generally hydrophobic S3 subsite located at the entrance of the SmCB1 active site cleft (Figure 3). Hydrophobic interactions are established with Leu139 and/or Ile145 (Figure 4B).

The N-terminal portion of the inhibitors represents the P4 position, which differs in the side chains of isoleucine (in gallinamide A and **1**) or phenylalanine (in **6**). The P4 is flipped out of the active site and is not oriented toward the S4 subsite (Figure 3B,C). This subsite is not well-defined in SmCB1 and other cathepsins B and has not yet been structurally described in available ligand-bound complexes. This is in line with the finding of low preferences for P4 substituents in human cathepsin B substrates.^48−50^ Furthermore, the P4 position exhibits the highest flexibility in all three inhibitors as evidenced by the B-factors (Figure S1), consistent with the conformational variability of the N-terminal segment of gallinamides recently reported.^27^ Not surprisingly, P4 is not involved in significant interactions with SmCB1, except for a hydrophobic interaction of the P4 phenylalanine of **6**, which is rotated to approach Leu139 in the S3 subsite (Figures 3C and 4B). The observed crystallographic conformation of P4 in gallinamides is likely affected by their analogous crystal packing through contacts made with the symmetry-related SmCB1 molecule.

### Computational Analysis of Inhibitor Positions Contributing to Gallinamide Binding

**Figure 5:**
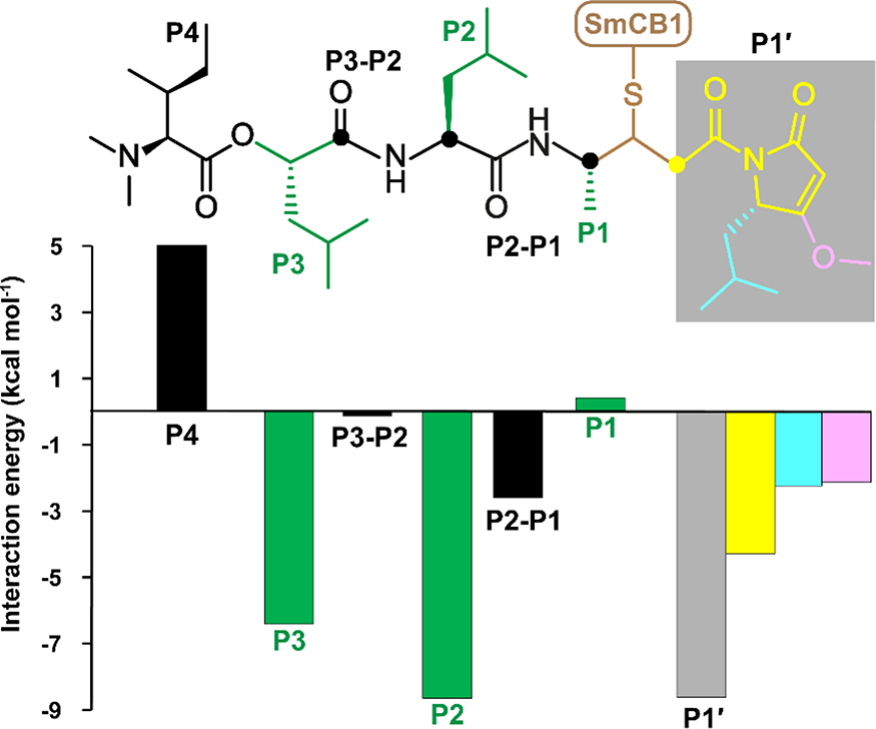
Energy contributions by each of the individual gallinamide positions that bind to SmCB1. The interaction “free” energy was determined using quantum chemical calculations on the crystallographic complex of SmCB1 with the gallinamide analog **1**. The inhibitor structure was fragmented into the individual segments (alternating black and green), including the positions P4–P1′ and backbone parts P3–P2 and P2–P1. The P1′ segment (gray box) was decomposed into three fragments, including the MMP group part (yellow) and its substituents (cyan, magenta). The contribution of the P1–P1′ segment, which forms a covalent bond with catalytic Cys100 (brown), was not calculated. The graph is labeled and color coded as in the inhibitor structure and presents the interaction “free” energy of the segments, which compares “free” energies of the separated solvated protein and ligand (segment) with the “free” energy of the solvated protein–ligand (segment) complex.

Quantum chemical calculations were carried out on the crystallographic complex of **1**, a gallinamide analog with high inhibitory potency and bioactivity, to determine the interaction “free” energy of the inhibitor in the binding subsites of SmCB1. Figure 5 shows the noncovalent interaction “free” energies of the individual side-chain and main-chain segments of the inhibitor structure in the P4–P1′ positions. The analysis revealed that the largest favorable contributions come from the P1′ and P2 positions (−8.6 kcal mol^–1^ each), followed by the P3 position (−6.4 kcal mol^–1^). The last two contributions are attributed to leucine side chains occupying the predominantly hydrophobic S2 and S3 pockets that form hydrophobic interactions. The P1′ segment was further decomposed into three fragments, including the MMP group and its substituents involved in several types of interactions (Figure 4), and their interaction “free” energies demonstrated that all contribute favorably. In contrast to the segments containing side chains P2 and P3, the adjacent main-chain segments P3–P2 and P2–P1 have rather smaller favorable contributions of −0.14 and −2.6 kcal mol^–1^, respectively. The most unfavorable segment (5.0 kcal mol^–1^) of the ligand is the position P4, which is flexible and exposed to solvent in the crystal structure. The absence of close contacts with the active site suggests that the positive interaction “free” energy of the P4 isoleucine arises from an unfavorable change of solvation “free” energy upon binding.

## Discussion

Gallinamides derived from gallinamide A, a natural peptidic metabolite of marine cyanobacteria, have been reported to inhibit cathepsin L-type cysteine proteases, such as human cathepsin L, falcipain, and cruzain.^24,26−29,51^ In this study, we show that gallinamides inhibit cathepsin B-type cysteine proteases, as demonstrated for SmCB1 from the flatworm parasite *S. mansoni*. Gallinamides are a new chemotype with potent antischistosomal activity.

SmCB1 is the most abundant digestive protease in the gut of *S. mansoni* and a promising target for antischistosomal drugs.^12,15,18^ We profiled the enzymatic activities of component cathepsins in the digestive proteolytic network of *S. mansoni* and showed that cathepsin B activity can be selectively and potently inhibited by gallinamide A. Furthermore, in *S. mansoni* exposed *ex vivo* to gallinamides, the activity of SmCB1 was dramatically reduced compared with untreated worms. Finally, using an alkyne-tagged gallinamide A and click chemistry to generate a fluorescent ABP probe, we specifically detected SmCB1 in worm extracts. The activity-based imaging, combined with the protease activity profiling, identifies SmCB1 as the primary target for gallinamide A and its analogs in adult *S. mansoni*.

We screened gallinamide A and 18 analogs to identify a SAR for the target protease and identified the most bioactive compounds against two developmental stages of the parasite that infect the mammalian host, namely, schistosomula and adults. Against SmCB1, seven potent inhibitors with *k*_2nd_ values >10^3^ M^–1^·s^–1^ were found, with gallinamide A being the most potent (a *k*_2nd_ value of 6644 M^–1^ s^–1^ and an IC_50_ value of 44 nM). Bioactivity screening identified potent antischistosomal analogs, including gallinamide A. Further derivation of the scaffold to enhance potency and bioactivity will be performed in the future, including the testing of potential cotargets such as digestive schistosomal proteases from the cathepsin L group (SmCL1–SmCL3).^13,14^ A comparison of the current gallinamides with previously reported peptidomimetic vinyl sulfones and nitriles^15,19,20^ and small-molecule semicarbazones, thiosemicarbazones, and hydrazones^52,53^ shows that inhibition potency against SmCB1 is less than the subnanomolar previously measured;^19^ nonetheless, the antischistosomal activity of gallinamides is noteworthy. The severity of the phenotypic changes in the parasite in the low micromolar range makes gallinamides one of the more effective chemotypes among the antischistosomal peptidomimetics and small molecules investigated to date that are against protease and nonprotease targets.^15,19,20,39,44,52−55^

To describe the binding mode of gallinamides in the active site of SmCB1, we solved high-resolution crystal structures of gallinamide A and two analogs that were potent inhibitors of the target and cidal to the parasite. Based on those crystallographic data, quantum chemical calculations were performed to investigate the contributions of the individual positions of the gallinamides in the binding subsites of SmCB1. These studies revealed the various elements of the gallinamide structure that are of particular interest in the design of future SmCB1 inhibitors. The P4 position is exposed to solvent and does not form significant interactions with the enzyme to favorably contribute to the binding energy. The high flexibility of P4 is supported by an ester linkage between P4 and P3, which we found to be important for bioactivity. Appropriate substitutions in P4 may help to target the S4 subsite, the interactions of which have not yet been structurally described in SmCB1 and other cathepsins B. The P1 position of gallinamide A contains a slightly energetically unfavorable alanine side chain, and we propose exploring residues with extended side chains, such as homophenylalanine, to improved binding, as reported for other SmCB1 inhibitors.^15,19^ The P1′ position contains the MMP ring with two substituents, the methoxy group and R1′ substituent, for which the R configuration was preferred. There is a spatial relationship between the conformations of these substituents and His181 located on the occluding loop of SmCB1. Our crystallographic and computational analyses suggest that the spatial orientation of the methoxy group can be employed to introduce new interactions with the occluding loop (e.g., through a carboxyl group that would bind His181^15,46,47,56^) to design more potent and selective gallinamides targeting SmCB1.

## Conclusions

We show that gallinamides are potent inhibitors of the SmCB1 drug target in *S. mansoni* and effective antischistosomal compounds, as tested against two developmental stages of the parasite that infect the mammalian host. We show for the first time that the bioactivity of gallinamides is associated with inhibition of a cathepsin B-type cysteine protease. Based on the SAR, and the crystallographic and quantum chemical characterizations of the inhibition of SmCB1 by the gallinamides, we proposed future directions for their rational design as potential chemotherapeutic agents to treat schistosomiasis.

## Methods

### Materials

Gallinamide A and its derivatives (Table 2) were synthesized and purified as previously described, and the compounds were of at least 95% purity.^28^ All compounds passed the PAINS filter using a false positive remover.^57^ Specific polyclonal antibodies against SmCB1 were raised in rabbit,^45^ and the immunoglobulin fraction was purified from the serum on a HiTrap Protein A column (Cytiva) according to the manufacturer’s protocol.

### Production and Purification of Recombinant SmCB1

A nonglycosylated mutant of the SmCB1 zymogen (Uniprot accession Q8MNY2) was expressed in the yeast *Pichia pastoris* using the pPICZαA vector and chromatographically purified as described previously.^45,58^ All purification steps were performed in the presence of 2 mM DTT and 1 mM EDTA under an argon atmosphere to prevent the active site cysteine residue from oxidation.

### Preparation of SmCB1–Inhibitor Complex

The zymogen form of SmCB1 (0.8–1.4 mg mL^–1^) was activated with *S. mansoni* legumain^59^ and simultaneously inhibited by a 4-fold molar excess of inhibitor (gallinamide A, **1**, or **6**) in 50 mM sodium acetate, pH 5.0, containing 20 mM cysteine and 1 mM EDTA for 10–18 h at room temperature under an argon atmosphere, as described previously.^58,60^ Protease inhibition was monitored using a kinetic assay with the fluorogenic substrate Cbz–Phe–Arg–AMC.^15^ The inhibitor complexes were chromatographed on an FPLC MonoS column (Cytiva) in 25 mM MES, pH 6.3, containing 2.5 mM DTT and 1 mM EDTA,^58^ buffer-exchanged into 10 mM sodium acetate, pH 5.5 and concentrated using Amicon Ultracel-10k centrifugal units (Millipore) to a final protein concentration of 7.4, 5.5, and 2.4 mg mL^–1^ for SmCB1–gallinamide A, SmCB1–**1**, and SmCB1–**6** complexes, respectively.

### Protein Crystallization and Data Collection

Crystals of the SmCB1 complexes with the inhibitor (gallinamide A, **1**, or **6**) were obtained by vapor diffusion in hanging drops using streak seeding. Drops consisted of (i) 1.5 μL of the complex of **1** or **6** and 0.75 μL of reservoir solution or (ii) 1 μL of the complex of gallinamide A and 1 μL of reservoir solution. The drops were equilibrated over 0.5 mL of reservoir solution at 5 (for gallinamide A and **1**) or 18 °C (for **6**). The reservoir solutions consisted of 0.2 M ammonium acetate and 0.1 mM sodium citrate, including (i) 30% (w/v) PEG 1500, pH 6.0 and pH 6.1 for gallinamide A and **6**, respectively, or (ii) 30% (w/v) PEG 3350, pH 6.0 for **1**. Protein concentrations of the stock solutions were 7.4, 5.5, and 2.4 mg mL^–1^ for the complexes of gallinamide A, **1**, and **6**, respectively. The obtained needle-shaped crystals were flash-cooled by plunging them into liquid nitrogen with cryoprotection (30% (w/v) PEG 300 in the reservoir solution). Diffraction data were collected at 100 K on beamline MX 14.1 at the BESSY electron-storage ring (Berlin, Germany)^61^ and processed using the XDS suite of programs.^62^ Crystal parameters and data collection statistics are given in Table S5.

### Structure Determination, Refinement, and Analysis

The structures of the SmCB1–gallinamide complexes were determined by molecular replacement with the program Molrep^63^ using the structure of mature SmCB1 (PDB code: 4I07)^45^ as a search model. Model refinement was performed with the program REFMAC 5.8.0257 from the CCP4 package,^64^ interspersed with manual adjustments using Coot.^65^ Anisotropic refinement of the atomic displacement parameters (ADPs, B-factors) was included in the refinement protocol for the gallinamide A complex. The geometric restraints for the ligands were constructed with the program Libcheck,^64^ using inhibitors optimized by quantum mechanics with the programs Turbomole 7.3^66^ and Cuby4^67^ by means of the DFT-D3/B3LYP/DZVP method^68^ combined with the COSMO2^69^ implicit solvent model. The quality of the final model was validated with Molprobity,^70^ and the final refinement statistics are given in Table S5. Atomic coordinates and structure factors were deposited in the Protein Data Bank with accession codes 8CC2, 8CCU, and 8CD9 for the SmCB1 complexes with gallinamide A, **1**, and **6**, respectively. The gallinamide A, **1**, and **6** molecules were modeled with occupancy factors of 0.6, 0.8, and 1.0, respectively, into generally well-defined electron density. Inhibitor interactions were analyzed using the programs CONTACT^64^ and PLIP.^71^ The distance cutoffs were set to 3.3 Å for hydrogen bonds and 4.1 Å for contacts. Hydrophobic interactions were determined using PLIP with a 4.1 Å distance limit between two hydrophobic atoms defined as carbon atoms neighboring carbon or hydrogen atoms. The conserved water-bridging molecules, shown as W1 and W2 in Figure 3, have the residue identifiers 512 and 674 for gallinamide A, 512 and 580 for **1**, 558 and 618 for **6**. All figures showing structural presentations were prepared with the program PyMOL 1.4 (Schrödinger).

### Computational Methods

The crystal structures of the SmCB1–gallinamide complexes were used to calculate the interaction “free” energy contributions of **1** and the relative “free” energies of the dual conformations of **6**. Hydrogen atoms were added to the crystallographic complexes using the software AMBER 14^72^ and PyMOL 1.8 and relaxed by annealing from 1500 to 0 K at the MM level in AMBER 14. The FF14SB force field was used for the protein, and the GAFF force field was used for the ligand. The cooling runs that utilized the Berendsen thermostat were 10 ps long with 1 fs steps. The crystallographic water molecules 512, 580, 611, 615, and 646 in the SmCB1–**1** complex were used to define the ligand environment. The positions of the inhibitor, water molecules, and hydrogen atoms were optimized in AMBER 14, implying the IGB7 implicit solvent model. All water molecules were removed from the SmCB1–**6** complex, and the positions of His181, inhibitor, and hydrogen atoms were optimized as above. To calculate the interaction “free” energy of the individual positions, the inhibitor was fragmented as presented in Figure 5. Cut bonds were capped with hydrogen atoms. The backbone segments (P2–P1 and P3–P2) were represented by CH_3_–NH–CO–H peptide bond fragments. The reactive vinyl moiety forming a covalent bond with catalytic Cys100 was not included in the calculations. The interaction and relative “free” energies were computed using the semiempirical quantum mechanical method PM6-D3H4X^73^ in combination with the COSMO2^69^ implicit solvent model using MOPAC2016^74^ and Cuby4^67^ software.

### Phenotypic Assays with *S. mansoni* Schistosomula and Adults

*S. mansoni* (NMRI strain) is maintained by cycling between *Biomphalaria glabrata* snails and Golden Syrian hamsters.^18,42^ Vertebrate animal use is supported under a protocol approved by UC San Diego′s Institutional Animal Care and Use Committee. The protocol complies with United States federal regulations regarding the care and use of laboratory animals: Public Law 99-158, the Health Research Extension Act, and Public Law 99-198, the Animal Welfare Act, which is regulated by USDA, APHIS, CFR, Title 9, Parts 1, 2, and 3.

Phenotypic screens employed *S. mansoni* newly transformed schistosomula (NTS) and adults and were carried out as described previously.^18−20,42^ NTS were prepared by mechanically transforming infective larvae (cercariae).^40,42^ NTS (200–300 parasites) were incubated in flat-bottomed 96-well plates in 200 μL of Basch Medium 169 containing 5% (v/v) FBS, 100 U mL^–1^ penicillin and 100 μg mL^–1^ streptomycin (complete Basch medium)^75^ at 5% CO_2_ and 37 °C.^76,77^ Inhibitors were added at final concentrations of 1 and 10 μM, and changes in phenotypes were observed under an inverted microscope every 24 h for up to 72 h.

For adult schistosomes, screens were performed in 24-well plates containing four male worms and one to two female worms per well in a final volume of 2 mL of complete Basch medium. Compounds were added at the final concentrations between 1 and 10 μM. Incubations were maintained for 48 h at 37 °C under 5% CO_2_. Afterward, the adult worms were collected, washed in RPMI 1640 medium, and used for soluble protein extract preparation.

A constrained nomenclature of “descriptors”^39,42^ was used to record the multiple and dynamic changes in movement, shape, and translucence that the schistosome parasite is capable of (Table S1 for NTS and Table S3 for adults). These descriptors were then converted into an ordinal “severity score” system ranging from 0 (no effect) to 4 (maximum effect), which allows for the relative comparison of compound effects.

### Preparation of Schistosome Extract

Soluble protein extracts (0.2–3.0 mg protein mL^–1^) of mixed sex *S. mansoni* adults were prepared by homogenization in 0.5 M sodium acetate, pH 5.5, containing 1% (w/v) CHAPS and 0.1 M NaCl on ice. The extract was cleared by centrifugation (16,000*g* at 4 °C for 10 min), ultrafiltered using a 0.22 μm Ultrafree-MC device (Millipore), and stored at −80 °C.

### Protease Activity Assays

Proteolytic activities in *S. mansoni* adult extracts were measured in a continuous kinetic assay using the following peptidyl fluorogenic substrates (Bachem) or internally quenched FRET substrates (IOCB): 50 μM Cbz–Phe–Arg–AMC for cathepsins B and L,^13,15^ 50 μM Cbz–Arg–Arg–AMC for endopeptidase activity of cathepsin B,^33,34^ 3.2 μM Abz–Phe–Arg–Val–Nph–OH for carboxydipeptidase activity of cathepsin B,^15^ 50 μM Gly–Arg–AMC for cathepsin C,^78,79^ 50 μM Cbz–Ala–Ala–Asn–AMC for legumain,^80^ 50 μM Cbz–Gly–Pro–AMC for prolyl oligopeptidase,^44^ and 50 μM Abz–Lys–Pro–Ala–Glu–Phe–Nph–Ala–Leu for cathepsin D.^81^ Measurements were performed at 37 °C in 96-well microplates in total volume of 100 μL. Parasite extract (0.2–5 μg of proteins) was preincubated for 10 min in 80 μL of 0.1 M sodium acetate, pH 4.0 (for cathepsin D) or pH 5.5 (for cysteine proteases), containing 1 mM EDTA, 0.1% (w/v) PEG 1500, and 2.5 mM dithiothreitol (for cysteine proteases), or 0.1 M Tris-HCl, pH 8.0, containing 10 mM E-64 and 1 mM EDTA (for prolyl oligopeptidase), followed by the addition of substrate (20 μL in the same buffer). Substrate hydrolysis was measured continuously using an Infinite M1000 microplate reader (Tecan) at excitation and emission wavelengths of 360/465 nm for AMC substrates and 320/420 nm for FRET substrates. To authenticate the measured activities, an aliquot of the extract was preincubated (15 min at 35 °C) in the assay buffer in the presence or absence of the following inhibitors: 1 μM E-64 for cathepsins B/L,^82^ 1 μM CA-074 for cathepsin B,^83^ 1 μM Ala–Hph–VS–Ph for cathepsin C,^84^ 1 μM Cbz–Ala–Ala–(aza–Asn)–CH=CH–COOEt (Aza–N–11a),^85^ and 10 μM pepstatin for cathepsin D^86^ before substrate addition.

### Inhibition Assays

Inhibition measurements were performed in duplicate in 96-well microplates (100 μL assay volume) at 37 °C. SmCB1 (40 pM) was added to a mixture of the fluorogenic substrate Cbz–Phe–Arg–AMC (25 μM) and an inhibitor (0–100 μM) in 0.1 M sodium acetate, pH 5.5, containing 2.5 mM dithiothreitol and 0.01% (w/v) BRIJ 35. Substrate hydrolysis was monitored in an Infinite M1000 microplate reader (Tecan) at excitation and emission wavelengths of 360 and 465 nm, respectively, for up to 150 min. An observed first-order rate constant, *k*_obs_, was calculated at each inhibitor concentration by fitting the progress curve to the equation *P* = *v_i_*/*k*_obs_(1 – exp(−*k*_obs_*t*)) + *d* where *P* is the product formation, *t* is the reaction time, *v_i_* is the initial velocity, and *d* is the offset. The *k*_obs_ values varied linearly with the inhibitor concentration indicating that the dependence of *k*_obs_ on the inhibitor concentration is nonsaturating. This kinetic mechanism does not allow determination of the individual *k*_inact_ and *K*_*i*_ parameters. The second-order rate constant, *k*_2nd_, (and SE value) was determined by fitting the linear equation *k*_obs_ = (*k*_2nd_[*I*])/(1 + [*S*]/*K*_m_), where [*S*] is the substrate concentration, [*I*] is the inhibitor concentration, and *K*_M_ is the Michaelis–Menten constant. The *K*_M_ value determined for SmCB1 was 25 μM. In all assay systems, the final concentration of DMSO did not exceed 1.5% (v/v). To determine IC_50_ values, SmCB1 (40 pM) was preincubated with the inhibitor (0–100 μM) for 30 min under assay conditions as described above, followed by the addition of the fluorogenic substrate Cbz–Phe–Arg–AMC (20 μM). Substrate hydrolysis was monitored for 30 min in a microplate reader as described above. The IC_50_ values were determined by nonlinear regression using GraFit software (Erithacus Software).

### Imaging of SmCB1 with Activity-Based Probe 5 and Immunolocalization

Recombinant SmCB1 (0.5 μg) or *S. mansoni* adult extract (7 μg protein) was incubated with 10 μM activity-based probe **5** (containing the alkyne tag) in 10 μL of 0.1 M sodium acetate, pH 5.0, containing 5 mM DTT at 37 °C for 1 h. Competitive labeling was performed after preincubation of SmCB1 or protein extract with the 10 μM E-64 for 15 min at 37 °C. For Cu(1)-catalyzed azide–alkyne cycloaddition, the reaction mixtures were diluted to 50 μL with water and incubated at room temperature for 30 min after the addition of 0.25 μL 5 mM of Alexa Fluor 647-azide (Thermofisher), 0.5 μL of 5 mM tris[(1-benzyl-1*H*-1,2,3-triazol-4-yl)methyl]amine (TBTA, Sigma), 1 μL of 25 mM sodium ascorbate, and 1 μL of 50 mM CuSO_4_. Sodium ascorbate and CuSO_4_ solutions were freshly prepared. The labeled proteins were precipitated with acetone, resolved by SDS-PAGE, and visualized using a Typhoon RGB imager (GE Healthcare Life Sciences) via excitation at 635 nm and emission at 660 nm (long pass filter) or transferred onto a PVDF membrane. The membrane was blocked for 1 h with 10% (w/v) nonfat dry milk and 1% (w/v) polyvinylpyrrolidone in 0.1 M Tris-HCl, pH 7.5, containing 150 mM NaCl and 0.1% (v/v) Tween (TTBS), washed with TTBS, and incubated for 1 h with rabbit polyclonal anti-SmCB1 antibody diluted 1:100 in TTBS. After washing with TTBS, the membrane was incubated with goat HRP-conjugated antirabbit IgG antibody (Sigma-Aldrich) at a dilution of 1:10 000 in TTBS for 1 h, developed with Immobilon Forte Western HRP substrate (Merck), and imaged using an ImageQuant LAS 4000 biomolecular imager (GE Healthcare Life Sciences).

## Supporting Information Available

(Table S1) Phenotypic effects and antischistosomal activity of gallinamides tested against *S. mansoni* schistosomula, (Table S2) physicochemical parameters of gallinamide A and its derivatives, (Table S3) phenotypic effects of gallinamides against *ex vivo**S. mansoni* adults, (Table S4) cytotoxicity of gallinamides, (Table S5) X-ray data collection and refinement statistics, (Table S6) list of contacts formed between SmCB1 and gallinamide inhibitors, (Table S7) atom–atom contacts between gallinamide A and SmCB1, (Table S8) atom–atom contacts between inhibitor **1** and SmCB1, (Table S9) atom–atom contacts between inhibitor **6** and SmCB1, (Table S10) computational analysis of dual conformations of the crystallographic structure of inhibitor **6** in the SmCB1 active site, and (Figure S1) analysis of the conformational flexibility of three gallinamide inhibitors in the SmCB1 active site (PDF)

## Accession Codes

Atomic coordinates and experimental structure factors have been deposited in the Protein Data Bank with accession code 8CC2, 8CCU, and 8CD9 for SmCB1–gallinamide A, SmCB1–**1**, and SmCB1–**6** complexes, respectively.

## Abbreviations

Abz: aminobenzoic acid
AMC: aminomethylcoumarin
Cbz: benzyloxycarbonyl
NTS: newly transformed schistosomula
Nph: 4-nitrophenylalanine
RMSD: root-mean-square deviation
SmCB1: cathepsin B1 from *S. mansoni*
SmPOP: prolyl oligopeptidase from *S. mansoni*
