# Supplementary material for: Nature-Inspired Gallinamides Are Potent Antischistosomal Agents: Inhibition of the Cathepsin B1 Protease Target and Binding Mode Analysis
Source: ACS Infect Dis. 2024 May 17;10(6):1935–48. doi: 10.1021/acsinfecdis.3c00589 (PMC11184554; doi:10.1021/acsinfecdis.3c00589)
Supplement: Supplementary file 1 — id3c00589_si_001.pdf [file id3c00589_si_001.pdf]

Supporting Information

Nature-Inspired Gallinamides Are Potent Antischistosomal Agents: Inhibition of the cathepsin B1 Protease Target and Binding Mode Analysis

Petra Spiwoková[1,2], Martin Horn[1], Jindřich Fanfrlík[1], Adéla Jílková[1], Pavla Fajtová[1,3], Adrian Leontovyč[1], Radka Houštecká[1,4], Lucia Bieliková[1,4], Jiří Brynda[1], Marta Chanová[5], Helena Mertlíková-Kaiserová[1], Eduardo J. E. Caro-Diaz[6], Jehad Almaliti[3,6], Nelly El-Sakkary[3], William H. Gerwick[3,6], Conor R. Caffrey[3], and Michael Mareš[1*]

[1]Institute of Organic Chemistry and Biochemistry of the Czech Academy of Sciences, Flemingovo n. 2, 16610 Prague 6, Czech Republic
[2]Department of Biochemistry and Microbiology, University of Chemistry and Technology, Technická 5, Prague 6 166 28, Czech Republic
[3]Center for Discovery and Innovation in Parasitic Diseases, Skaggs School of Pharmacy and Pharmaceutical Sciences, University of California, San Diego, La Jolla, California 92093, United States
[4]First Faculty of Medicine, Charles University, Kateřinská 32, 12108 Praha 2, Czech Republic
[5]Institute of Immunology and Microbiology, First Faculty of Medicine, Charles University and General University Hospital in Prague, Studničkova 2028/7, 12800 Prague 2, Czech Republic
[6]Scripps Institution of Oceanography, University of California, San Diego, La Jolla, California 92093, United States
[*]Corresponding author: e-mail: mares@uochb.cas.cz.

Contents

Table S1. Phenotypic effects and antischistosomal activity of gallinamides tested against *S. mansoni* schistosomula.
Table S2. Physicochemical parameters of gallinamide A and its derivatives.
Table S3. Phenotypic effects and antischistosomal activity of gallinamides tested against *S. mansoni* adults.
Table S4. Cytotoxicity of gallinamides.
Table S5. X-ray data collection and refinement statistics.
Table S6. List of contacts formed between SmCB1 and gallinamide inhibitors.
Table S7. Atom–atom contacts between gallinamide A and SmCB1.
Table S8. Atom–atom contacts between inhibitor **1** and SmCB1.
Table S9. Atom–atom contacts between inhibitor **6** and SmCB1.
Table S10. Computational analysis of dual conformations of the crystallographic structure of inhibitor **6** in the SmCB1 active site.
Figure S1. Analysis of the conformational flexibility of three gallinamide inhibitors in the SmCB1 active site.

Supplementary tables

Table S1. Phenotypic effects of gallinamides against *S. mansoni* schistosomula.

	Phenotype descriptors[a]						Severity scores[b]					
**Compound (μM)**	**1**			**10**			**1**			**10**		
**Time (h)**	**24**	**48**	**72**	**24**	**48**	**72**	**24**	**48**	**72**	**24**	**48**	**72**
**Gallinamide A**	R	Dark, R	Deg	Deg	**D**	**D**	1	2	4	4	4	4
**1**	R, unc	Deg	Deg	Deg	**D**	**D**	2	4	4	4	4	4
**2**	N	R	Deg	Deg	Deg	**D**	0	1	4	4	4	4
**3**	N	N	Dark, R	N	Dark, R	Dark, R	0	0	2	0	2	2
**4**	N	N	Deg	unc	Deg	Deg	0	0	4	1	4	4
**5**	N	Deg	**D**	Dark	Deg	**D**	0	4	4	1	4	4
**6**	R, unc	Deg	**D**	**D**	**D**	**D**	2	4	4	4	4	4
**7**	N	Dark, R, S	Deg	Dark	Deg	**D**	0	3	4	1	4	4
**8**	Dark	Deg	D	Dark, R, S	**D**	**D**	1	4	4	3	4	4
**9**	R	Dark, R	Deg	Dark, R, S	**D**	**D**	1	2	4	3	4	4
**10**	N	Dark, R	Deg	N	Deg	**D**	0	2	4	0	4	4
**11**	R	Dark, R, S	Deg	Dark	Deg	**D**	1	3	4	1	4	4
**12**	Dark	Dark, R	**D**	Dark, R, S	**D**	**D**	1	2	4	3	4	4
**13**	N	Dark	Deg	Dark, R	Deg	**D**	0	2	4	2	4	4
**14**	N	Dark, R, unc	**D**	Dark	Deg	**D**	0	3	4	1	4	4
**15**	N	Dark, R, unc	**D**	Dark, R	Deg	**D**	0	3	3	2	4	4
**16**	N	N	N	N	Dark, R	Dark, R	0	0	0	0	2	2
**17**	N	N	Deg	Dark	Deg	**D**	0	0	4	1	4	4
**18**	N	N	N	Dark	Deg	**D**	0	0	0	1	4	4

[a]Phenotypic changes in newly transformed schistosomula (NTS) of *S. mansoni* induced by 1 and 10 μM compounds were recorded daily for three days. Phenotypes are reported using the following descriptors: N, normal; R, rounded; S, slow; unc, uncoordinated; Dark, dark, color altered from normal; Deg, degenerated; D, dead. [b]Each descriptor is assigned a value of 1, except for Deg and **D**, which are given the maximum score of 4. Values are then added to yield a severity score ranging from 0 (no effect) to 4 (the most severe), as described previously. [1-3]

Table S2. Physicochemical parameters of gallinamide A and its derivatives.

Compound	MW	nON[a]	nOHNH[b]	logP	TPSA (A^2)[c]	Rotatable Bonds[d]
GallinamideA	592.8	11	2	3.53	134.4	20
**1**	634.9	11	2	4.34	134.4	22
**2**	578.7	11	2	3.18	134.4	19
**3**	605.8	12	4	2.57	163.0	21
**4**	591.8	11	3	3.17	137.1	20
**5**	658.9	11	2	4.96	134.4	25
**6**	702.9	11	2	4.99	134.4	22
**7**	745.0	11	2	5.77	134.4	24
**8**	668.9	11	2	4.52	134.4	22
**9**	668.9	11	2	4.58	134.4	22
**10**	710.9	11	2	5.37	134.4	24
**11**	696.9	11	2	5.05	134.4	23
**12**	702.9	11	2	4.96	134.4	22
**13**	779.0	11	2	5.99	134.4	24
**14**	745.0	11	2	5.77	134.4	24
**15**	745.0	11	2	5.77	134.4	24
**16**	594.8	11	2	3.79	134.4	21
**17**	779.0	11	2	5.99	134.4	24
**18**	745.0	11	2	5.77	134.4	24

[a]Number of hydrogen bond acceptors. [b]Number of hydrogen bond donors. [c]Topological polar surface area. [d]Number of rotatable bonds. Physicochemical parameters were determined from https://www.molinspiration.com/cgi-bin/properties and ALOGPS2.1 software from http://www.vcclab.org/web/alogps/.

Table S3. Phenotypic effects of gallinamides against *ex vivo S. mansoni* adults.

Phenotype descriptors[a]																
**Time (h)**	**2**				**6**				**24**				**48**			
**Compound (µM)**	**1**	**2**	**5**	**10**	**1**	**2**	**5**	**10**	**1**	**2**	**5**	**10**	**1**	**2**	**5**	**10**
**Gallinamide A**	N	N	N	N	N	N	N	N	N	N	N	unc	N	S, dark	S, dark	S, on sides, dark
**1**	N	N	N	N	N	N	N	unc	N	N	N	S, on sides, teg dam	N	unc, dark	unc, dark	S, on sides, teg dam
**6**	N	N	unc	unc, on sides	N	N	unc	unc, on sides	N	N	S, on sides, teg dam	S, on sides, teg dam	N	dark	unc, on sides, teg dam	S, on sides, teg dam
**9**	N	N	unc	unc	N	N	unc	unc, on sides	N	N	unc, Dark	S, on sides, teg dam	N	unc, dark	unc, on sides, teg dam	S, on sides, teg dam

[a]Phenotypic changes in adult *S. mansoni* induced by compounds at given concentrations were recorded after 2, 6, 24 and 48 h. Phenotypes are reported as the following descriptors: N, normal; S, slowed motility; Unc, uncoordinated movements; Dark, dark - color altered from normal; on sides, male worms do not adhere to dish with ventral sucker; teg dam, outer surface (tegument of the worm) is damaged; deg, degenerated. Each descriptor is assigned a value of 1, except for deg and teg dam, which are given the maximum score of 4. Values are then added to yield a severity score ranging from 0 (no effect) to 4 (the most severe), as described previously. [1, 4, 5]

Table S4. Cytotoxicity of gallinamides.

Cytotoxicity of gallinamide A and compounds **1**, **6**, and **9** was tested with four human cell lines and expressed as % viability vs. untreated cells. Cells were treated with the indicated compound concentrations for 72 h (A) or with 10 µM compounds for the indicated time (B) and assayed using CellTiter Glo 2.0 (Promega) according to the manufacturer's protocol. Cytotoxicity was expressed as percent viability compared to untreated control cells; the standard deviation of the mean of quadruplicate readings is shown. Cell line abbreviations: HL-60, human promyelocytic leukemia; HeLa, human cervical carcinoma; HepG2, human hepatocellular carcinoma; NHDF, normal human dermal fibroblasts.

A

Cell line viability after 72 h (%)								
**Cell line**	**HL-60**		**HeLa**		**HepG2**		**NHDF**	
**Compound**	**1 µM**	**10 µM**	**1 µM**	**10 µM**	**1 µM**	**10 µM**	**1 µM**	**10 µM**
**Gallinamide A**	100 ± 6	73 ± 5	96 ± 4	88 ± 6	96 ± 3	94 ± 11	105 ± 6	94 ± 94
**1**	99 ± 7	89 ± 4	96 ± 1	107 ± 8	104 ± 4	106 ± 5	102 ± 2	98 ± 97
**6**	100 ± 5	11 ± 0	93 ± 4	13 ± 1	104 ± 3	13 ± 1	99 ± 3	13 ± 13
**9**	99 ± 4	73 ± 1	93 ± 2	99 ± 6	94 ± 6	79 ± 3	100 ± 0	75 ± 76

B

Cell line viability (%) after treatment with 10 µM compound												
**Cell line**	**HL-60**			**HeLa**			**HepG2**			**NHDF**		
**Time (h)**	**24**	**48**	**72**	**24**	**48**	**72**	**24**	**48**	**72**	**24**	**48**	**72**
**Gallinamide A**	99 ± 5	77 ± 3	73 ± 5	77 ± 4	73 ± 3	88 ± 6	93 ± 6	98 ± 7	94 ± 11	80 ± 2	89 ± 3	94 ± 94
**1**	103 ± 6	89 ± 7	89 ± 4	88 ± 3	100 ± 2	107 ± 8	91 ± 3	91 ± 8	106 ± 5	93 ± 2	100 ± 6	98 ± 97
**6**	31 ± 1	11 ± 2	11 ± 0	45 ± 1	13 ± 1	13 ± 1	61 ± 1	14 ± 1	13 ± 1	100 ± 2	35 ± 3	13 ± 13
**9**	92 ± 6	85 ± 10	73 ± 1	95 ± 6	98 ± 5	99 ± 6	97 ± 3	77 ± 3	79 ± 3	84 ± 7	93 ± 4	75 ± 76

**% viability**	90+	80+	70+	60+	50+	40+	30+	20+	10+	0+

Table S5. X-ray data collection and refinement statistics.

SmCB1–inhibitor complex[a]	SmCB1–Gallinamide A	SmCB1–Cpd.1	SmCB1–Cpd.6
**Data collection statistics**			
Wavelength (Å)	0.918	0.918	0.918
Temperature (K)	100	100	100
Space group	$P2_12_12_1$	$P2_12_12_1$	$P2_12_12_1$
a, b, c (Å)	33.18, 79.21, 90.63	33.10, 79.07, 90.63	33.06, 78.87, 90.15
α, β, γ (°)	90.00, 90.00, 90.00	90.00, 90.00, 90.00	90.00, 90.00, 90.00
Resolution (Å)	50.00–1.20 (1.27–1.20)	50.00–1.60 (1.70–1.60)	50.00–1.55 (1.64–1.55)
Number of unique reflections	75696 (11996)	32051 (4938)	35093 (5478)
Multiplicity	6.6 (6.6)	5.9 (3.6)	6.8 (6.7)
Completeness (%)	99.6 (98.7)	99.4 (96.5)	99.2 (97.1)
R_{merge}[b] (%)	8.7 (140.4)	11.6 (94.4)	15.4 (180.1)
Average I/σ (I)	11.79 (1.19)	10.65 (1.22)	9.54 (0.97)
$CC_{1/2}$ (%)	99.9 (48.9)	99.7 (48.2)	99.7 (47.7)
Wilson B (Å^2)	17.2	23.1	23.9
**Refinement statistics**			
Resolution range (Å)	45.36–1.20 (1.23–1.20)	45.29–1.60 (1.64–1.60)	39.44–1.55 (1.59–1.55)
Number of reflections in working set	74185 (5337)	30418 (2121)	33338 (2333)
Number of reflections in test set	1511 (106)	1601 (112)	1755 (123)
R value[c] (%)	13.5 (37.4)	16.0 (31.7)	17.8 (37.9)
R_{free} value[d] (%)	16.5 (36.3)	19.4 (37.4)	21.6 (36.6)
Number of molecules in AU[e]	1	1	1
Number of atoms in AU[e] protein/inhibitor/solvent	2090/42/273	2087/45/264	2088/69/211
Average ADP[f] for protein/inhibitor/solvent (Å^2)	14.3/22.3/29.3	17.5/33.3/25.2	19.9/37.8/27.3
RMSD bond length (Å)	0.012	0.013	0.012
RMSD bond angle (°)	1.66	1.70	1.70
Ramachandran plot statistics[g]			
Favored regions (%)	96.5	96.9	96.9
Allowed regions (%)	3.5	3.1	3.1
PDB ID	8CC2	8CCU	8CD9
Ligand ID	GN9	UX9	UA9

[a]Numbers in the parentheses refer to the highest-resolution shell.

[b]$R_{merge}=100\sum_{hkl}\sum_i|I_i(hkl) - \langle I(hkl)\rangle|/\sum_{hkl}\sum_i I_i(hkl)$, where $I_i(hkl)$ is an individual intensity of the ith observation of the reflection *hkl* and ‹I(*hkl*)› is the average intensity of the reflection *hkl* with summation over all data.

[c]R value = $||F_o| - |F_c||/|F_o|$, where F_o and F_c are the observed and calculated structure factors, respectively.

[d]R_{free} is equivalent to the R value but is calculated for up to 5% of the reflections chosen at random and omitted from the refinement process. [6]

[e]AU, asymmetric unit.

[f]ADP, atomic displacement parameter, formally B-factor.

[g]As determined by Molprobity. [7]

Table S6. List of contacts formed between SmCB1 and gallinamide inhibitors.
The analysis of protein-inhibitor contacts between the SmCB1 active site and the inhibitors was performed using the program CONTACT. [8] The distance cutoffs were set to 4.1 Å for all contacts and 3.3 Å for hydrogen bonds. Fragmentation of the inhibitors into the positions P4 to P1' is depicted in black/red above the table; distinguishing substituents are highlighted in color (magenta, green and cyan for gallinamide A, **1** and **6**, respectively). The SmCB1 residues interacting with the individual inhibitor position (P3 to P1´) are specified. The C atom of inhibitors forming a covalent bond with the enzyme is included in the analysis (P1). For each SmCB1 residue, the total number of contacts is given (∑), and the individual types of contacts are indicated, including carbon–carbon (C-C), carbon–heteroatom (C-h), and heteroatom–heteroatom (h-h) contacts. Hydrogen bonds (Hb) are indicated; residues forming Hb are in bold. For compound **6,** data are shown for two alternative conformations (A and B) of the P1' position.

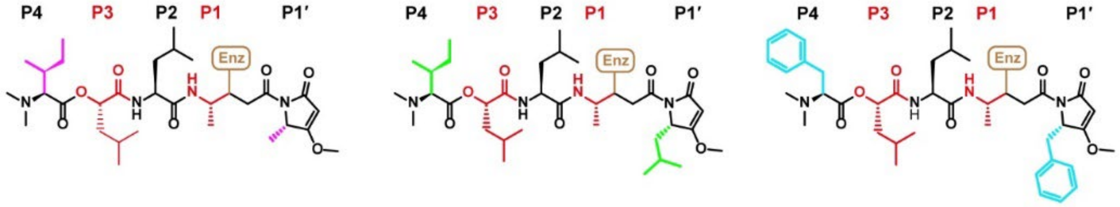

Position	Gallinamide A Residue	Contacts ∑	C-C/ C-h/ h-h	Hb	Cpd.1 Residue	Contacts ∑	C-C/ C-h/ h-h	Hb	Cpd.6 Residue	Contacts ∑	C-C/ C-h/ h-h Conf.A	C-C/ C-h/ h-h Conf.B	Hb
P1'	**Gln94**	2	0/1/1	1	**Gln94**	3	0/2/1	1	**Gln94**	2/4	0/1/1	0/2/2	1
									Ser95	0/2		0/2/0	
									Arg96	0/1		1/0/0	
									Cys97	0/1		0/1/0	
	Gly98	1	0/1/0		Gly98	1	0/1/0		Gly98	4/0	2/2/0		
	Cys100	3	1/2/0		Cys100	4	1/2/1		Cys100	3/3	1/2/0	1/2/0	
									Cys141	1/0	0/1/0		
									His180	0/3		1/2/0	
	His181	1	0/1/0		His181	2	0/1/1		His181	0/2		0/1/1	
					Ile193	1	1/0/0		Ile193	2/2	2/0/0	2/0/0	
	Val247	2	0/2/0		Val247	1	0/1/0		Val247	1/0	0/1/0		
	Leu252	1	1/0/0										
	Leu267	1	1/0/0		Leu267	1	1/0/0		Leu267	0/1		1/0/0	
	Gly269	2	0/1/1		Gly269	3	0/2/1		Gly269	2/1	0/1/1	0/1/0	
	His270	5	0/4/1		His270	7	2/5/0		His270	5/5	2/3/0	3/2/0	
									Trp292	4/1	4/0/0	0/0/1	
P1	Gln94	1	0/1/0		Gln94	1	0/1/0						
	Gly98	8	5/3/0		Gly98	8	5/3/0		Gly98	8	5/3/0		
	Cys100	3	0/2/1		Cys100	3	0/2/1		Cys100	3	0/2/1		
	**Gly269**	3	0/2/1	1	**Gly269**	3	0/2/1	1	**Gly269**	4	0/3/1		1
					His270	1	0/1/0		His270	1	0/1/0		
P2	Gly98	1	0/0/1		Gly98	1	0/0/1		Gly98	1	0/0/1		
	Cys100	2	0/1/1		Cys100	2	0/1/1		Cys100	2	0/1/1		
	Trp101	1	0/1/0		Trp101	3	0/3/0		Trp101	2	0/2/0		
	Gly143	2	0/2/0		Gly143	3	1/2/0		Gly143	3	1/2/0		
	**Gly144**	10	0/7/3	2	**Gly144**	8	0/5/3	2	**Gly144**	11	0/7/4		2
	Leu146	2	2/0/0		Leu146	3	3/0/0		Leu146	2	2/0/0		
	Gly269	5	3/2/0		Gly269	5	3/2/0		Gly269	5	3/2/0		
	His270	3	1/2/0		His270	3	1/2/0		His270	3	1/2/0		
	Glu316	4	1/3/0		Glu316	5	1/4/0		Glu316	5	1/4/0		
P3	Gly138	1	1/0/0		Gly138	1	1/0/0		Gly138	1	1/0/0		
	Leu139	3	2/1/0		Leu139	2	1/1/0		Leu139	3	2/1/0		
	Gly143	3	2/1/0		Gly143	4	2/2/0		Gly143	4	3/1/0		
	Gly144	8	3/5/0		Gly144	7	3/4/0		Gly144	5	0/5/0		
	Ile145	1	1/0/0		Ile145	1	1/0/0						
P4									Leu139	2	2/0/0		
	Glu142	1	0/1/0		Glu142	1	0/1/0		Glu142	2	0/2/0		
									Gly268	1	1/0/0		

Table S7. Atom–atom contacts between gallinamide A and SmCB1.

The numbering of the inhibitor atoms is according to the PDB. Pairs of enzyme and inhibitor atoms forming contacts are listed. Atoms forming hydrophobic interactions and hydrogen bonds are highlighted in red and blue, respectively. See Table S6 for details.

Gallinamide A

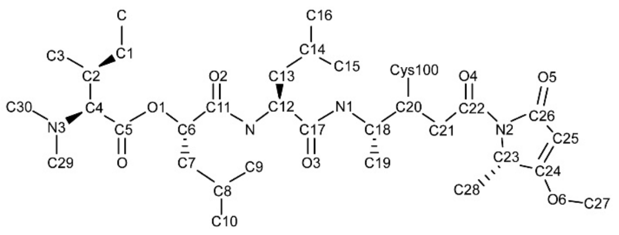

Position	Enzyme residue	Contact count	Enzyme residue atom<br>Ligand atom									
	Gln94	2	**NE2<br>O4**	NE2<br>C22								
	Gln98	1	CA<br>O4									
	Cys100	3	CB<br>C21	SG<br>C21	SG<br>C22							
	His181	1	NE2<br>C27									
**P1'**	Val247	2	CG1<br>O5	CG2<br>O5								
	Leu252	1	CD1<br>C27									
	Leu267	1	CD1<br>C27									
	Gly269	2	O<br>O5	O<br>C5								
	His270	5	CA<br>O5	CB<br>O5	CG<br>O5	ND1<br>O5	ND1<br>C5					
	Gln94	1	NE2<br>C20									
**P1**	Gly98	8	CA<br>C18	C<br>C18	O<br>C18	CA<br>C19	O<br>C19	CA<br>C20	C<br>C20	O<br>C20		
	Cys100	3	SG<br>C18	SG<br>C19	SG<br>N1							
	Gly269	3	O<br>C18	C<br>N1	**O<br>N1**							
	Gly98	1	O<br>O3									
	Cys100	2	SG<br>C17	SG<br>O3								
	Trp101	1	CD1<br>O3									
	Gly143	2	CA<br>O3	C<br>O3								
**P2**	Gly144	10	O<br>C13	O<br>C14	N<br>C17	O<br>C17	O<br>C12	**O<br>N**	**N<br>O3**	CA<br>O3	C<br>O3	O<br>O3
	Leu146	2	**CG<br>C15**	**CD2<br>C15**								
	Gly269	5	CA<br>C16	C<br>C16	O<br>C17	C<br>C12	O<br>C12					
	His270	3	N<br>C16	C<br>C16	O<br>C16							
	Glu316	4	CD<br>C15	OE2<br>C15	OE1<br>C16	OE2<br>C14						
	Gly138	1	CA<br>C10									
	Leu139	3	N<br>C10	**CG<br>C10**	**CD1<br>C10**							
**P3**	Gly143	3	C<br>C7	C<br>C10	O<br>C10							
	Gly144	8	O<br>C11	O<br>C6	N<br>C7	CA<br>C7	C<br>C7	O<br>C7	N<br>C10	CA<br>C10		
	Ile145	1	**CG1<br>C9**									
**P4**	Glu142	1	O<br>C30									

Table S8. Atom–atom contacts between inhibitor 1 and SmCB1.

The numbering of the inhibitor atoms is according to the PDB. Pairs of enzyme and inhibitor atoms forming contacts are listed. Atoms forming hydrophobic interactions and hydrogen bonds are highlighted in red and blue, respectively. See Table S6 for details.

Cpd.1

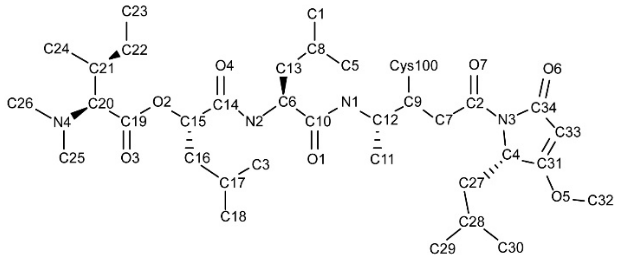

Position	Enzyme residue	Contact count	Enzyme residue atom<br>Ligand atom							
P1'	Gln94	3	CD<br>O7	**NE2**<br>**O7**	NE2<br>C2					
	Gly98	1	CA<br>O7							
	Cys100	4	SG<br>O7	CB<br>C7	SG<br>C7	SG<br>C2				
	His181	2	NE2<br>O5	NE2<br>C32						
	Ile193	1	**CD1**<br>**C30**							
	Val247	1	CG2<br>O6							
	Leu267	1	CD1<br>C32							
	Gly269	3	C<br>O6	O<br>O6	O<br>C7					
	His270	7	CA<br>O6	CB<br>O6	CG<br>O6	CA<br>C7	CG<br>C7	ND1<br>C7	ND1<br>C2	
P1	Gln94	1	NE2<br>C9							
	Gly98	8	CA<br>C12	C<br>C12	O<br>C12	CA<br>C11	O<br>C11	CA<br>C9	C<br>C9	O<br>C9
	Cys100	3	SG<br>N1	SG<br>C12	SG<br>C11					
	Gly269	3	C<br>N1	**O**<br>**N1**	O<br>C12					
	His270	1	ND1<br>C9							
P2	Gly98	1	O<br>O1							
	Cys100	2	SG<br>O1	SG<br>C10						
	Trp101	3	CB<br>O1	CG<br>O1	CD1<br>O1					
	Gly143	3	CA<br>O1	C<br>O1	CA<br>C10					
	Gly144	8	**N**<br>**O1**	CA<br>O1	C<br>O1	O<br>O1	O<br>C6	O<br>C13	N<br>C10	**O**<br>**N2**
	Leu146	3	**CG**<br>**C1**	**CD1**<br>**C1**	**CD2**<br>**C1**					
	Gly269	5	C<br>C6	O<br>C6	CA<br>C5	C<br>C5	O<br>C10			
	His270	3	N<br>C5	C<br>C5	O<br>C5					
	Glu316	5	OE2<br>C8	OE1<br>C5	CD<br>C1	OE1<br>C1	OE2<br>C1			
P3	Gly138	1	CA<br>C3							
	Leu139	2	N<br>C3	**CG**<br>**C3**						
	Gly143	4	C<br>C3	O<br>C3	C<br>C16	O<br>C16				
	Gly144	7	CA<br>C3	N<br>C16	CA<br>C16	C<br>C16	O<br>C16	O<br>C15	O<br>C14	
	Ile145	1	**CG1**<br>**C3**							
P4	Glu142	1	O<br>C26							

Table S9. Atom–atom contacts between inhibitor 6 and SmCB1.
The numbering of the inhibitor atoms is according to the PDB. Pairs of enzyme and inhibitor atoms forming contacts (CC, contact count) are listed. Atoms forming hydrophobic interactions and hydrogen bonds are highlighted in red and blue, respectively. See Table S6 for details.

Cpd.6

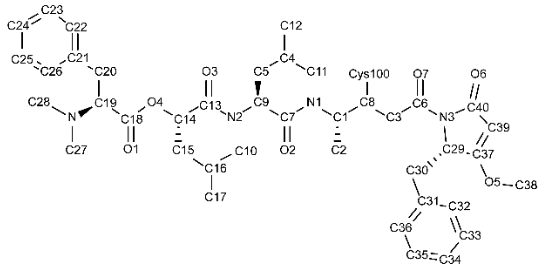

Double conformation of primed part of the Cpd.6

Position	Conformation A							Conformation B						
	Residue	CC	Residue atom<br>Ligand atom					Residue	CC	Residue atom<br>Ligand atom				
	Gln94	2	**NE2**<br>**O7**	NE2<br>C6				Gln94	4	CD<br>O7	OE1<br>O7	**NE2**<br>**O7**	NE2<br>C6	
	Gly98	4	CA<br>O7	N<br>C36	CA<br>C36	CA<br>C35		Ser95	2	O<br>C35	O<br>C34			
								Arg96	1	CA<br>C34				
								Cys97	1	N<br>C34				
	Cys100	3	CB<br>C3	SG<br>C3	SG<br>C6			Cys100	3	SG<br>C6	CB<br>C3	SG<br>C3		
P1'								His180	3	ND1<br>C36	ND1<br>C35	CE1<br>C35		
	Cys141	1	O<br>C35					His181	2	NE2<br>O5	NE2<br>C38			
	Ile193	2	**CG2**<br>**C36**	**CG2**<br>**C35**				Ile193	2	**CG2**<br>**C33**	**CG2**<br>**C32**			
	Val247	1	CG2<br>O6					Leu267	1	CD1<br>C38				
	Gly269	2	O<br>O6	O<br>C3				Gly269	1	O<br>C3				
	His270	5	CB<br>O6	CG<br>O6	CA<br>C3	CG<br>C3	ND1<br>C3	His270	5	ND1<br>C6	CA<br>C3	CG<br>C3	ND1<br>C3	CE1<br>C3
	Trp292	4	CZ2<br>C40	CH2<br>C39	CZ2<br>C39	CZ2<br>C37		Trp292	1	NE1<br>O7				

Single conformation of non-primed part of theCpd.6

Position	Residue	CC	Residue atom<br>Ligand atom										
	Gly98	8	CA<br>C2	C<br>C2	O<br>C2	CA<br>C1	C<br>C1	O<br>C1	C<br>C8	O<br>C8			
P1	Cys100	3	SG<br>N1	SG<br>C2	SG<br>C1								
	Gly269	4	C<br>N1	**O**<br>**N1**	O<br>C1	O<br>C8							
	His270	1	ND1<br>C8										
	Gly98	1	O<br>O2										
	Cys100	2	SG<br>O2	SG<br>C7									
	Trp101	2	CB<br>O2	CD1<br>O2									
	Gly143	3	CA<br>O2	C<br>O2	CA<br>C7								
P2	Gly144	11	**N**<br>**O2**	CA<br>O2	C<br>O2	O<br>O2	N<br>N2	**O**<br>**N2**	O<br>C9	N<br>C7	O<br>C7	O<br>C5	O<br>C4
	Leu146	2	**CG**<br>**C12**	**CD2**<br>**C12**									
	Gly269	5	C<br>C9	O<br>C9	O<br>C7	CA<br>C11	C<br>C11						
	His270	3	N<br>C11	C<br>C11	O<br>C11								
	Glu316	5	OE2<br>C4	CD<br>C12	OE2<br>C12	OE1<br>C11	OE1<br>C11						
P3	Gly138	1	CA<br>C17										
	Leu139	3	N<br>C17	**CG**<br>**C17**	**CD1**<br>**C17**								

	Gly143	4	C<br>C17	O<br>C17	CA<br>C15	C<br>C15	
	Gly144	5	N<br>C17	N<br>C15	O<br>C15	O<br>C14	O<br>C13
**P4**	Leu139	2	**CD1**<br>**C24**	**CD1**<br>**C23**			
	Glu142	2	O<br>C23	O<br>C22			
	Gly268	1	CA<br>C28				

Table S10. Computational analysis of dual conformations of the crystallographic structure of inhibitor 6 in the SmCB1 active site.

The alternative conformations of three flexible segments were analyzed using quantum chemical calculations, including the methoxy group (OMe) and the phenylalanine side chain (Phe) on the MMP ring of the inhibitor in P1' and His181 of the enzyme. The table shows in bold five favorable combinations with relative 'free' energies (ΔG') below 5 kcal mol^{-1}, of which combinations 4 and 5 (marked with an asterisk) represent the minimal set of complementary conformations that explain the observed electron density maps. The figure shows the modeled conformations (A in magenta, B in cyan) in sticks representation; heteroatoms have a standard color coding.

Combination	Phe (inh)	Inh-OMe (inh)	His181 (enz)	ΔG´ (kcal mol^{-1})
**1**	**A**	**B**	**A**	**0.0**
**2**	**A**	**B**	**B**	**1.3**
**3**	**B**	**B**	**A**	**1.9**
**4***	**A**	**A**	**A**	**3.8**
**5***	**B**	**B**	**B**	**3.9**
6	B	A	A	5.4
7	B	A	B	11.9
8	A	A	B	14.2

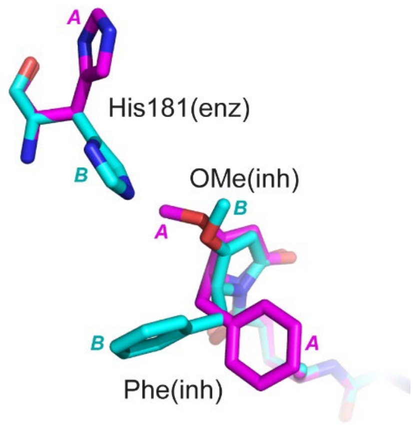

Supplementary figures

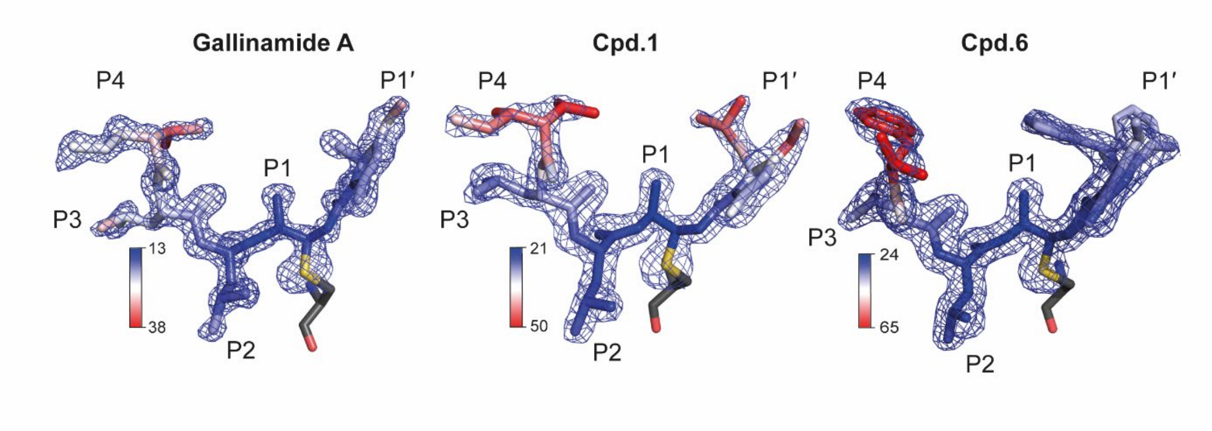

Figure S1. Analysis of the conformational flexibility of three gallinamide inhibitors in the SmCB1 active site.

The inhibitors and the covalently linked catalytic cysteine residue are shown in sticks representation. The $2F_o-F_c$ electron density maps are contoured at 0.7 σ. Structures of inhibitors are colored according to atomic B-factor values (given in Å^2), from blue (low) to red (high). The B-factor range is different for each inhibitor. The highest B-factors, indicating conformational flexibility, are observed in the terminal parts of the inhibitors (the P4 and P1′ positions). Two different P1′ conformations of compound **6** are presented. The cysteine residue is colored gray (heteroatoms have a standard color coding: O, red; N, blue; S, yellow); the electron density map of its main chain was omitted for clarity. The crystal structure resolutions and inhibitor occupancy factors are as follows: gallinamide A – 1.20 Å and 0.6, compound **1** – 1.60 Å and 0.8, compound **6** – 1.55 Å and 1 (0.5 for each P1′ conformation). The orientation of each inhibitor is set for clarity of presentation.

References

(1) Long, T.; Neitz, R. J.; Beasley, R.; Kalyanaraman, C.; Suzuki, B. M.; Jacobson, M. P.; Dissous, C.; McKerrow, J. H.; Drewry, D. H.; Zuercher, W. J.; Singh, R.; Caffrey, C. R. Structure-Bioactivity Relationship for Benzimidazole Thiophene Inhibitors of Polo-Like Kinase 1 (PLK1), a Potential Drug Target in *Schistosoma mansoni*. *PLoS Negl. Trop. Dis.* **2016**, *10* (1), e0004356. DOI: 10.1371/journal.pntd.0004356.

(2) Jílková, A.; Horn, M.; Fanfrlík, J.; Küppers, J.; Pachl, P.; Řezáčová, P.; Lepšík, M.; Fajtová, P.; Rubešová, P.; Chanová, M.; Caffrey, C. R.; Gütschow, M.; Mareš, M. Azanitrile Inhibitors of the SmCB1 Protease Target Are Lethal to *Schistosoma mansoni*: Structural and Mechanistic Insights into Chemotype Reactivity. *ACS Infect. Dis.* **2021**, *7* (1), 189-201. DOI: 10.1021/acsinfecdis.0c00644.

(3) Jílková, A.; Rubešová, P.; Fanfrlík, J.; Fajtová, P.; Řezáčová, P.; Brynda, J.; Lepšík, M.; Mertlíková-Kaiserová, H.; Emal, C. D.; Renslo, A. R.; Roush, W. R.; Horn, M.; Caffrey, C. R.; Mareš, M. Druggable Hot Spots in the Schistosomiasis Cathepsin B1 Target Identified by Functional and Binding Mode Analysis of Potent Vinyl Sulfone Inhibitors. *ACS Infect. Dis.* **2021**, *7* (5), 1077-1088. DOI: 10.1021/acsinfecdis.0c00501.

(4) Monti, L.; Cornec, A. S.; Oukoloff, K.; Kovalevich, J.; Prijs, K.; Alle, T.; Brunden, K. R.; Smith, A. B., 3rd; El-Sakkary, N.; Liu, L. J.; Syed, A.; Skinner, D. E.; Ballatore, C.; Caffrey, C. R. Congeners Derived from Microtubule-Active Phenylpyrimidines Produce a Potent and Long-Lasting Paralysis of *Schistosoma mansoni* In Vitro. *ACS Infect. Dis.* **2021**, *7* (5), 1089-1103. DOI: 10.1021/acsinfecdis.0c00508.

(5) Abdulla, M. H.; Ruelas, D. S.; Wolff, B.; Snedecor, J.; Lim, K. C.; Xu, F.; Renslo, A. R.; Williams, J.; McKerrow, J. H.; Caffrey, C. R. Drug discovery for schistosomiasis: hit and lead compounds identified in a library of known drugs by medium-throughput phenotypic screening. *PLoS Negl. Trop. Dis.* **2009**, *3* (7), e478. DOI: 10.1371/journal.pntd.0000478.

(6) Brünger, A. T. Free R value: a novel statistical quantity for assessing the accuracy of crystal structures. *Nature* **1992**, *355* (6359), 472-475. DOI: 10.1038/355472a0.

(7) Lovell, S. C.; Davis, I. W.; Arendall, W. B., III; de Bakker, P. I.; Word, J. M.; Prisant, M. G.; Richardson, J. S.; Richardson, D. C. Structure validation by Calpha geometry: phi,psi and Cbeta deviation. *Proteins: Struct. Funct. Bioinform.* **2003**, *50* (3), 437-450. DOI: 10.1002/prot.10286.

(8) Winn, M. D.; Ballard, C. C.; Cowtan, K. D.; Dodson, E. J.; Emsley, P.; Evans, P. R.; Keegan, R. M.; Krissinel, E. B.; Leslie, A. G.; McCoy, A. Overview of the CCP4 suite and current developments. *Acta Crystallogr. D Biol. Crystallogr.* **2011**, *67* (4), 235-242. DOI: 10.1107/s0907444910045749.
